# Predictive Models of Gene Regulation from High-Throughput Epigenomics Data

**DOI:** 10.1155/2012/284786

**Published:** 2012-08-13

**Authors:** Sonja Althammer, Amadís Pagès, Eduardo Eyras

**Affiliations:** ^1^Computational Genomics, Universitat Pompeu Fabra, Dr. Aiguader 88, Barcelona, Spain; ^2^Institució Catalana de Recerca i Estudis Avançats (ICREA), Passeig Lluís Companys 23, Barcelona, Spain

## Abstract

The epigenetic regulation of gene expression involves multiple factors. The synergistic or antagonistic action of these factors has suggested the existence of an epigenetic code for gene regulation. Highthroughput sequencing (HTS) provides an opportunity to explore this code and to build quantitative models of gene regulation based on epigenetic differences between specific cellular conditions. We describe a new computational framework that facilitates the systematic integration of HTS epigenetic data. Our method relates epigenetic signals to expression by comparing two conditions. We show its effectiveness by building a model that predicts with high accuracy significant expression differences between two cell lines, using epigenetic data from the ENCODE project. Our analyses provide evidence for a degenerate epigenetic code, which involves multiple genic regions. In particular, signal changes at the 1st exon, 1st intron, and downstream of the polyadenylation site are found to associate strongly with expression regulation. Our analyses also show a different epigenetic code for intron-less and intron-containing genes. Our work provides a general methodology to do integrative analysis of epigenetic differences between cellular conditions that can be applied to other studies, like cell differentiation or carcinogenesis.

## 1. Introduction

DNA associates with histone proteins to conform the chromatin [1]. Histones generally carry posttranscriptional modifications in cells capable of modulating the expression of genes [2, 3]. For instance, there is a genome-wide relation between the histone 3 lysine 36 trimethylation (H3K36me3) and transcription activity [4, 5]. This and other epigenetic modifications are key to cellular differentiation [6] and their alterations have been associated to early stages of cellular transformation in tumors [7, 8]. The combinations of the histone modifications, which can have cooperative or opposed effects on the chromatin state, have been proposed to reflect a histone code that would determine the regulation of gene expression and the cell state [9]. High-throughput sequencing (HTS) technologies provide a very effective way to obtain information about the histone modification patterns at genome wide scale [10]. Efforts to integrate available genome-wide datasets about chromatin in various conditions are crucial towards improving our understanding of the role of epigenetics in gene regulation.

Recent publications have made progress in the definition of a histone code of gene expression by generating predictive models of transcriptional activity based on histone mark information [11–17]. They provide insights into possible mechanisms of regulation and a formal description of the postulated histone code [18, 19]. These methods generally relate the histone signals obtained from experiments of chromatin immunoprecipitation followed by HTS (ChIP-Seq) [20], with a read-out of the gene expression based on expression microarrays or HTS for mRNAs (RNA-Seq) [21]. In these approaches, the chromatin signal is generally represented in terms of read-counts or peak significance in the promoter and sometimes the gene body of genes. However, this analysis is generally based on one single condition or cell line. That is, they effectively compare the properties of different genes in a direct way, relying on the premise that signals in two different genes should be comparable, and the accuracy of their predictive model will be dependent on the accuracy of the estimation of the significance of the ChIP-Seq signals. However, genes present many variable properties, like number of introns or the presence of CpG islands in their promoter, which may affect these measurements. For instance, recent experiments show that the splicing machinery can recruit histone-modifying enzymes and influence the chromatin state, with the consequence that genes with introns tend to have higher levels of H3K36me3 signal [22]. Thus, the comparison of genes with and without introns is not straightforward. Additionally, various other factors may affect the local density of HTS signal [23]. For instance, the tag counts from an HTS experiment will be influenced by the chromatin structure of the DNA and by shearing effects [24–26], not all regions have the same mappability [27] and there is often a GC bias in the reads [28]. These issues will reflect on differences in coverage between regions, which will be even more exacerbated for the broad signals that are obtained for histone ChIP-Seq experiments. Control samples can partly alleviate this, but their effectiveness depends very much on the sequencing depth. Thus, HTS signals from two genes are not directly comparable in general.

Here, we propose a new method to measure epigenetic signals and to relate them to expression based on the comparison between two conditions. In our approach, the same genomic locus is compared between two conditions; hence, the predictive model describes changes of gene expression in terms of relative changes in epigenetic mark densities between two conditions or cell types. Significance of these changes is calculated taking the read density into account, thereby mitigating the confounding effects mentioned earlier. Additionally, unlike a previous method that has made pairwise comparisons of epigenetic data from cell lines [17], our method considers continuous changes of the epigenetic signal densities, rather than an on-off state description. Moreover, our framework provides greater flexibility than previous approaches for the generation of computational predictive models.

To illustrate our method, we have built a model of expression regulation from epigenetic changes using data from various ENCODE cell lines [29]. In order to extend this relation, we include additional epigenetic data not considered previously, namely, HTS of DNase I hypersensitive sites (DNase-Seq) [30] and DNA methylation data [31]. Our results show a different epigenetic code for expression for intron-less and intron-containing genes, being this difference more prominent in genes with low GC content around the transcription start site. Moreover, eliminating anti-sense transcription and overlapping promoters and tails from different genes, which has not been done before, the prediction accuracy improves considerably. Furthermore, the predictive model built from one pair of cell lines performs with high accuracy in a different pair. Finally, we are able to generate a minimal code for expression regulation between two cell lines that is generic enough to correctly predict the regulatory outcome of up to 70% transcripts from a different pair of cell lines.

## 2. Material and Methods

### 2.1. Genomic Annotations

For our analyses, we used the gene set from the 7th release of the GENCODE annotation (ftp://ftp.sanger.ac.uk/pub/gencode/release_7/gencode.v7.annotation.gtf.gz), which is based on the assembly GRCh37 (hg19) and is included in the ensembl release 62 [32]. All transcripts encoded at each gene loci and the genomic region defined by them, which we name transcript loci, were considered initially. Those transcript loci from chromosome M and of biotype “pseudogene” were removed for the analysis.

We separated transcript loci into four groups; according to whether they were intron-containing (IC) or intron-less (IL), and according to whether they had a promoter with high CpG (HCG) or low CpG (LCG) content. We classified transcripts as HCG if the region of 4 kb centred on the transcription start site (TSS) overlaps at least 200 bp with a CpG island, and LCG otherwise. CpG island annotations where obtained from the UCSC Table Browser (hg19) [33]. In order to obtain balanced sets for training and testing, an equal number of up- (Up) and down- (Dw) regulated transcripts were selected from each of the four groups. These groups were taken to be as large as possible, but such that the *P*-value of significance (Benjamini-Hochberg corrected) for the expression change for each transcript was smaller than 0.05. Furthermore, the same number of nonregulated (Nr) transcripts was selected. These are defined to have the highest *P*-values and sufficient expression, that is, the density of reads measured in RPKM (reads per kilobase per million mapped reads) as defined in [21] was greater than 1 in a cell line from the pair. With this, we obtained four different sets (Table 1). As part of our analyses, we also filtered overlapping transcript loci that would make ambiguous the assignment of the marks with the correct expression change. That is, we removed the loci from both strands when they were in any of the following configurations (Supplementary Figure 1, available at doi:10.1155/2012/284786):

- transcript loci that overlap in opposite strands,
- transcript loci whose promoters (2 kb) overlap in opposite strands,
- transcript loci whose tails (2 kb) overlap in opposite strands
- transcript loci with overlapping promoter (2 kb) and tail (2 kb) on the same strand,
- Overlapping transcript loci on the same strand but from different genes.

### 2.2. Datasets

We downloaded ChIP-Seq data for RNA Polymerase II (RNAPII), CCCTC-binding factor (CTCF) and various Histone marks (Table 2), data for DNase I hypersensitive sites (DNase-Seq), methylation data from reduced representation bisulfite sequencing (Methyl-RRBS) and RNA-Seq data from the ENCODE project (http://hgdownload.cse.ucsc.edu/goldenPath/hg19/encodeDCC/) for four cell lines: a chronic myelogenous leukemia line (K562), a lymphoblastoid line (GM12878), a human mammary epithelial line (HMEC), and a muscle myoblast line (HSMM, Table 2). We considered two pairs of comparisons, P1: K562 versus GM12878 and P2: HSMM versus HMEC. To further validate these results, we also considered a third comparison, K562 versus HSMM, P3. We selected experiments that were available in these four cell lines, except for RNAPII, which was only available in two of the selected cell lines. For all datasets, we used only reads that did not contain any uncalled bases (N). Moreover, for ChIP-Seq and DNase-Seq reads, we kept only reads with mapping quality greater than 30. The Methyl-RRBS data was filtered for positions covered by at least 10 reads. The mean methylation of a region was defined to be the proportion of methylated sites over the total number of probed sites in that region. Further, we obtained the RPKMs for the RNA-Seq data for the individual transcript loci directly from ENCODE public datasets (http://hgdownload-test.cse.ucsc.edu/goldenPath/hg19/encodeDCC//wgEncodeCshlLongRnaSeq/releaseLatest/).

For our analysis we considered for each transcript locus, a number of regions related to its exon-intron structure (Table 3). Subsequently, for each one of these regions and for each experimental dataset, the *z*-score for the enrichment was calculated between a pair of cell lines using Pyicos [34]. The calculation was based on 2 replicas in one condition (K562 or HSMM) and 1 replica in the other condition (GM12878 or HMEC). Further, pseudocounts and RPKM normalization were used (details in Supplementary Material). These *z*-scores constitute the set of attributes that were used for Machine-Learning (ML) analyses and corresponds to each region-experiment pair. As a control, random attributes were generated for each region by random sampling *z*-score values from all attributes for that region type. 

Unless otherwise stated, accuracies of the models were measured calculating the average area under the receiver operating characteristic (ROC) curve (AUC) for a 10-fold crossvalidation. A ROC curve relates the rates of true positives (TPs) and false positives (FPs) produced by the model. The larger the area described by the ROC curve (AUC) the better the overall accuracy of the model. AUC = 1 indicates a model that predicts no false positives and all true cases correctly, and AUC = 0.5 indicates that the model is equivalent to random. In 10-fold crossvalidation, the data is split into 10 subsets and 10 evaluations are carried out iteratively, where in each iteration 9 subsets (nine-tenths of the instances) are used for training and one subset for testing. This method ensures that all instances are used for the evaluation and the overall accuracy is averaged over the ten iterations, so that it represents the mean behaviour of the model.

### 2.3. Read Profiles around Gene Bodies

We calculated the average number of reads from different marks around gene bodies, by plotting the average number of reads in windows (−2000, +400) and (−400, +2000) around the TSS and pA, respectively. Reads from histone marks, RNAPII, and CTCF were extended to 300 bp in the 5′ to 3′ direction, whereas methyl-RRBS data was extended in either direction to 75 bp. Genes considered for the profiles were at least 400 bp long. We further filtered out pseudogenes and those loci that overlap each other (see above) and split the remaining ones into expressed (RPKM > 0) and nonexpressed (RPKM = 0) genes, resulting into 1202 IC and 1748 IL expressed genes, and 1385 IC and 746 IL nonexpressed genes. Supplementary Figures 2(A) and 2(B) show the profiles for IC and IL genes, whereas pseudogenes are shown in Supplementary Figure 2(C). Pseudogenes were also filtered for overlapping loci and for gene lengths shorter than 400 bp before they were split into 2277 IC and 3564 IL pseudogenes. 

## 3. Results and Discussion

### 3.1. A Framework for Integrative Epigenetic Studies

Our computational framework addresses three fundamental tasks in the process of acquiring knowledge: data-mining, data manipulation, and data analysis, and it is comprised of the following steps: (i) an analysis pipeline to systematically identify the changes in expression and epigenetic signals between two conditions in multiple genomic regions, (ii) an automatic way to store the results in a Biomart system [34] for easy querying and filtering and (iii) a connectivity to the application WEKA [35], to allow the application of Machine-Learning (ML) methods for creating predictive models of gene regulation. 

In order to relate epigenetic signals to expression regulation, our method measures signal changes between two conditions rather than the signal level in one single condition. With this methodology, relative changes of the epigenetic state can be related to each other or to the relative change of expression. By considering relative signal changes, biases from HTS are mitigated. To verify this, we checked whether selecting significant regions according to RPKM densities or *z*-scores from our method would be biased by the GC content. We, therefore, considered the top 10% of genes in terms of the H3K4me3 RPKM (K562) in the gene body and found a Spearman correlation of 0.34 with GC content. However, selecting the top 10% of genes according to absolute *z*-scores for H3K4me3, given by the comparison between K562 and GM12878, resulted in no correlation with GC content (Spearman 0.02). Thus, relating RPKM values to gene expression could result into false positives due to GC bias. When we repeated the same calculation on the 4 kb region centered on the TSS, none of the two measures, RPKMs or *z*-scores, showed a GC bias (correlation coefficient of −0.02 and 0.05, resp.). As H3K4me3 is mostly distributed around the TSS [10], we deduce that in this case the real signal obscures the bias, while in the gene body, where no strong signal for H3K4me3 is present, the bias dominates over the signal. 

We have developed an automatic pipeline that, given a set of regions and a number of high-throughput sequencing (HTS) datasets for two conditions, can systematically calculate the log-rate of change for each region and its significance in terms of a *z*-score (details in Supplementary File). The datasets used are accessible through a Biomart database at http://regulatorygenomics.upf.edu/group/pages/software/. We have modified Biomart so that datasets can also be exported as ARFF (attribute-relation file format), which can be uploaded directly into the WEKA system [35], a collection of open-source machine-learning algorithms for data-mining tasks, issued under the GNU General Public License. Our system thus provides the possibility of using own custom data to train models and evaluate different ML algorithms for the study of mechanisms of gene regulation.

**Figure 1:**
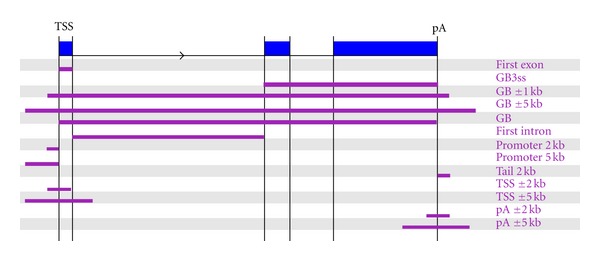
Graphical representation of the regions considered per transcript locus for the calculation of the different attributes. For detailed description of the regions see Table 3.

In order to illustrate the potential of our framework we analysed high-throughput sequencing (HTS) data from ENCODE [29] (Section 2). We started by systematically calculating the changes between cell lines in pair P1 (K562 versus GM12878) and in pair P2 (HSMM versus HMEC) for all the experiments in a variety of regions related to the transcript loci (Table 3). Most of the recently developed predictive methods use signals in the promoter region of genes or in a window around the transcription start site (TSS). We also included the gene body, as recent evidence suggests that the signal along this region will be informative as well [36]. Besides promoter, TSS, and gene body regions, we also include a region for the 1st exon, the 1st intron, and the gene body downstream of the 1st intron, which have been shown to contain relevant chromatin signatures for transcriptional regulation [22, 37, 38], and have not been used before in a predictive model. We further considered additional windows around and beyond the poly-adenylation site (pA), resulting in a total of 13 different regions (Table 3, Figure 1). Accordingly, for the two pairs of cell lines P1 and P2, we had a total of 13 × 12 = 156 and 13 × 11 = 143 (as RNAPII was not available for P2) attributes per transcript locus, respectively, where each attribute is defined by the *z*-score of the enrichment value between the two cell lines for a region-experiment pair.

As classification value, we used expression information from RNA-Seq experiments from ENCODE in the corresponding cell lines. For each pair of cell lines, we calculated the transcripts with significant increase (Up) or decrease (Dw) of expression. In order to build a predictive model of expression that can distinguish between either type of regulation (Up or Dw) and no change, we also considered nonregulated (Nr) transcripts, defined to have sufficient expression level and no significant change in expression between the same pair of cell lines (Section 2). 

Recent studies have shown that introns may influence the transcriptional regulation of genes [22, 38]. Therefore, we separated our transcripts sets according to whether they were intron-containing (IC) or intron-less (IL). Furthermore, several studies have highlighted that human promoters present different regulation according to their CG content [39–41]. Thus, we further split the sets according to whether a 4 kb region centered on the TSS overlaps with a CpG island or not, resulting in high CpG content (HCG) or low CpG content (LCG) sets (Section 2). Finally, in order to have a balanced set for training and testing, we selected from each type the same number of transcripts for each regulatory class (Table 1). 

### 3.2. A Generic Epigenetic Code for Gene Expression Regulation

Using the datasets processed as above, we built a highly accurate and generic predictive model of gene expression changes based on epigenetic data. We tried various ML models to predict the three possible classes, up (Up), down (Dw), and nonregulated (Nr), and we decided to use a random forest model [42], as it showed the best performance using 10-fold crossvalidation (data not shown).  Table 4 shows the accuracies of this model tested on intron-containing sets for various training conditions. Remarkably, we obtain a higher accuracy for the LCG set than for the HCG set (Table 4). Incidentally, CpG-related genes are quite often housekeeping genes [43], and this has been pointed out before as one of the reasons why predictive models perform differently on each set [44]. According to this, LCG transcripts should be more frequently associated to genes with differential expression (Up or Dw). This is confirmed in our analysis, as we found that the performance was always higher for the prediction of Up and Dw loci than for nonregulated transcripts (Table 4). For intron-less (IL) loci, we found the opposite behaviour, that is, HCG-IL has higher accuracy than LCG-IL (Supplementary Table 1). 

**Figure 2:**
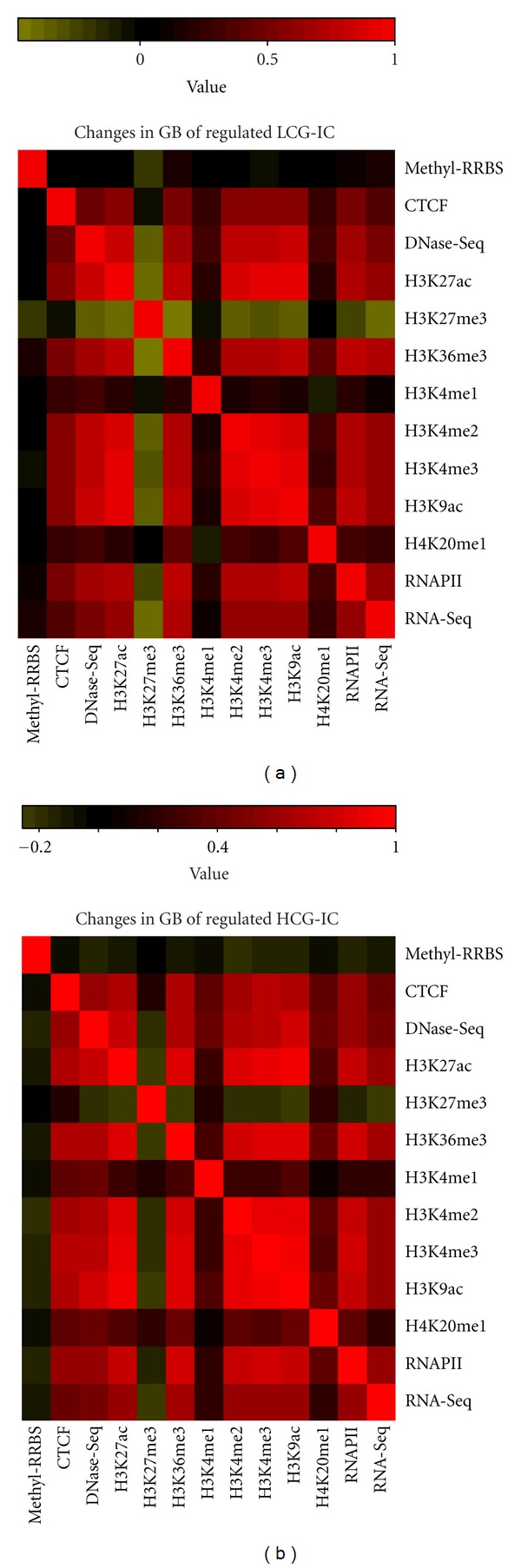
Pairwise correlations of marks and expression changes in gene bodies. Heatmaps are shown for regulated genes from the filtered intron-containing (IC) sets for low (LCG) (a) and high (HCG) (b) CpG promoters. The color represents the value of the Pearson correlation coefficient between the zscores for every pair of attributes. Both panels use the same scale, indicated above. For expression (RNA-Seq), the z-scores of the Up and Dw transcript loci were used to calculate the correlation.

Interestingly, training a model for the first pair with (Table 4(a), P1 (with RNAPII)) or without RNAPII data (Table 4(a), P1) yields very similar accuracy for all sets, which suggests that the information provided by RNAPII is redundant with the histone data for prediction. Indeed, looking at the pairwise correlations of all marks for P1, separated per region and per transcript set (Figure 2 and Supplementary Figure 3), we observe a high correlation of the *z*-scores for RNAPII with most of the other signals (H3K36me3, DNase-Seq, CTCF, H3K4me2, H3K9ac, H3K27ac, and H3K4me3).

With the aim of obtaining a minimal set of attributes that are sufficient to attain high prediction accuracy, we applied correlation-based feature selection (CFS) [45]. This method works by iteratively testing subsets of attributes, retaining those that best correlate with the class values (Up, Dw, or Nr) and removing those that have high redundancy. In this way, a minimal set of nonredundant attributes with optimal performance is selected. We applied CFS to the data from both pairs of cell lines and selected attributes that were selected in at least 80% of the validation rounds (Table 4(a), P1(CFS), and P2(CFS)). Interestingly, CFS provided attributes related to all the regions (Supplementary Table 2(A)), indicating that histone marks along all regions of the transcript locus may be relevant for regulation. Additionally, the prediction accuracy did not suffer, while the model is simplified by removing redundant attributes (Table 4(a), P1(CFS)).

With the aim of obtaining a generic epigenetic code of expression regulation, we decided to compare the attributes obtained from P1 with the attributes obtained for a second pair of cell lines (P2). Although CFS applied to both pairs, P1 and P2, yields a different set of optimal attributes, with only between 26% and 50% of coincidences between them (Supplementary Table 2), a model built on P2 with the attributes selected from P1 shows a high accuracy, which is comparable to the original model on P1 (Table 4(a) P2(CFS-P1)). That is, qualitatively, the attributes relevant for one pair of cell lines seem to be also relevant for the other one.

To test the generality of the model also in quantitative terms, that is, in terms of the actual numerical model, we applied directly on P2 the model built from P1. However, this test across pairs did not achieve an accuracy as high as before (Table 4(a), P1-on-P2 and P1(CFS)-on-P2). We hypothesized that the reduction of accuracy in the test across pairs could be due to differences in the homogeneity of cell lines, which would produce a very variable pattern of signals. Alternatively, this lack of reproducibility could stem from the overlap of the gene body, promoters or tails from transcript loci from different genes, especially in the opposite strand, which would make ambiguous the association of the epigenetic signal change to a specific expression change. Accordingly, we removed from the training set those transcripts loci where the signal in one region could not be unambiguously assigned (Section 2, Supplementary Figure 1), thereby generating filtered sets for training and testing (Table 1). Interestingly, after removing these cases we observe a consistent increase in the accuracy of the prediction in all groups (Table 4(b)), with 60–78% of the instances correctly classified (Table 5).

To further confirm our results, we considered a third pair comparison: K562 versus HSMM or Pair 3 (P3). Supplementary Table 3 shows that accuracies for P3 are similar to those in P1 and P2, with higher accuracy for LCG loci, as found for the other pairs. As before, the AUC increases when loci are filtered (Section 2). Moreover, as shown before for P1 and P2, after filtering, the model trained on P1 gives similar prediction accuracy when applied to P3. 

Despite the consistency of the models, there is still a fraction of instances that are incorrectly classified, that is, false positives. To understand why these instances cannot be correctly classified, we examined the the *z*-score distribution corresponding to the best separating attributes for up, down and nonregulated genes in LCG-IC and HCG-IC. Supplementary Figure 4 shows that the distributions of *z*-scores for the false positives in each class, Up, Dw, or Nr, show almost no differences between each other, as opposed to the true positives, which show a clear separation. Thus, there is a subset of loci where the changes in the studied marks are not sufficient to explain the change of expression.

We further explored whether the signals in one single region would be sufficient to predict the expression outcome. Accordingly, for each region, we selected the common attributes from pairs P1 and P2 with CFS score ≥80% (Supplementary Table 4). Interestingly, the marks selected for a single region give a prediction accuracy that is comparable to that obtained with attributes from all regions (Supplementary Table 5). The highest accuracy was achieved using gene body ±5 kb, which is not surprising as it overlaps all the other regions. Interestingly, the 2 kb region downstream of the pA turns out to have a high predictive power, achieving an AUC of 0.89 for upregulated IC-LCG transcripts based only on the signals for H3K27me3 and H3K36me3. Remarkably, one single mark in the region pA ±2 kb is enough to predict upregulated genes with high accuracy (AUC = 0.85 and 0.81 for Up in IC-LCG and IC-HCG transcripts, resp.). This is consistent with the enrichment of H3K36me3 found previously in a region around the pA for active genes [10]. As before, the models achieve higher AUCs for LCGs than for HCGs.

### 3.3. The Relative Contribution of Marks to the Epigenetic Code

**Figure 3:**
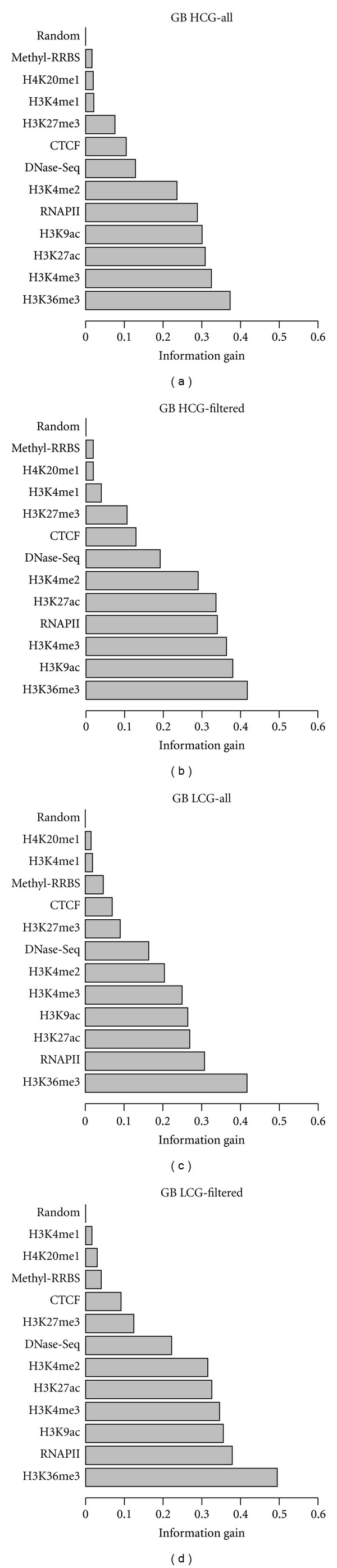
Information gain values measured for attributes in the gene body of intron-containing (IC) transcript loci, comparing before and after filtering loci according to overlap with transcripts from different genes (Section 2). Data is shown for high (HCG) and low (LCG) CpG promoters. Random attributes generated by random sampling *z*-score values from all attributes in a given region are shown as a control.

With the aim to find the most relevant attributes that appear to determine the regulation of expression, we calculated the information gain (IG) [46] for all attributes in the subsets HCG-IC and LCG-IC on pair P1 for the unfiltered and the filtered sets (Table 1). The higher the IG value, the better the attribute can separate the three classes: Up, Dw, and Nr. As a control, we generated random attributes for each region, obtained by random sampling *z*-score values from all attributes in that region. In Figure 3 and Supplementary Figure 5 we show how attributes rank in terms of IG within each region. Although the ranking is very similar before and after filtering transcript loci, we found an overall increase in IG values, indicating that the filtering step improves the specificity of the regulatory code. We found that for all subsets, H3K36me3 is the most informative attribute around the pA site and in gene body associated regions, whereas H3K27ac and H3K9ac are the most informative in the promoter region, which agrees with previous analyses [47]. These two acetylation marks are in fact among the most informative marks in the promoter, around the TSS and in 1st intron and 1st exon regions. Interestingly, H3K36me3 is more informative in the 1st intron than in the 1st exon, which agrees with recent results relating H3K36me3 with splicing of the first intron [22]. Although methylation data shows anticorrelation with expression change in the promoter of HCG loci (Supplementary Figure 6), we observe a modest contribution in the gene body to expression regulation (Figures 2 and 3).

**Figure 4:**
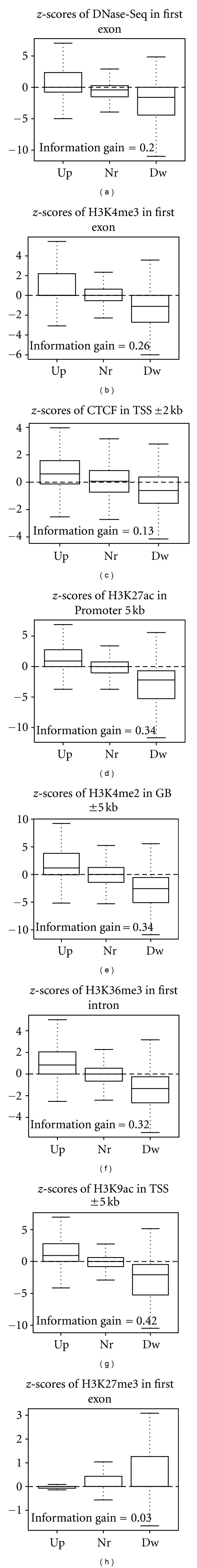
Distribution of *z*-scores for up- (Up), down- (Dw), and non- (Nr) regulated genes for the optimal attributes for each experiment, calculated by maximizing the Information Gain and minimizing the absolute value of the median for the *z*-score distribution of the Nr subset. The *y*-axis shows the *z*-score corresponding to the enrichment of the attribute. These distributions correspond to the set of LCG-IC loci of Pair1.

Although IG values determine how well an attribute separates the three sets, Up, Dw, and Nr, we would expect that attributes that most directly associate with expression changes should show no change for the Nr set. That is, we should expect that the enrichment *z*-scores for Nr should distribute around zero. Accordingly, we defined an attribute to be optimal if the absolute value of the median for the Nr distribution is smaller than 0.1 and the IG is greater than 0.05. If more than one attribute accomplish these thresholds, we considered the one with the highest IG value. Interestingly, this analysis shows that the optimal attributes for H3K36me3 and H3K4me3 correspond to the 1st intron and 1st exon, respectively (Figure 4), which could be related to their role in the coupling between splicing and transcription [22, 38]. Moreover, for H3K9ac and H3K27ac, the optimal attributes are the TSS-5 kb and Promoter-5 kb regions, respectively. DNase-Seq also presented the optimal distribution in the 1st exon, whereas CTCF and H3K4me2 were best in the GB-5 kb region. 

We did not find an optimal attribute for RNAPII. Although the attribute for the gene body has minimal median for the Nr distribution and largest IG (Supplementary Figure 7(A)), it shows an enrichment for Nr similar to the Up subset, which could be due to an excess of RNAPII reads in one of the cell lines (Supplementary Figure 7(B)). We also did not find optimal attributes for Methyl-RRBS, H3K4me1, and H4K20me1. For Methyl-RRBS, this is probably due to a large proportion of sites with reads but no methylation evidence (data not shown). The most informative region with minimal median for Nr for H3K4me1 indicates an enrichment of Up in GB ±5 kb but a distribution for Dw and Nr centered on zero, indicating an asymmetry in transcriptional activation. Although H4K20me1 has been related to silent chromatin [48], the most informative of the attributes showed almost no difference between Up, Dw, and Nr subsets. The absence of an optimal attribute for H3K4me1 in GB ±5 kb and for H4K20me1 in the 1st exon might be due to an unequal distribution of reads in K562 relative to GM12878, which does not occur for H3K27me3. Finally, even though we could not find an optimal attribute for H3K27me3, the *z*-score distributions for the 1st exon results into a clear trend that agrees with the anticorrelation of H3K27me3 and expression (Figure 4), despite the low IG (0.03): Up genes show almost no change, whereas Dw genes show the greatest enrichment, possibly indicating that there is an asymmetry in the pattern of this histone mark for silencing.

### 3.4. The Effect of Introns in the Epigenetic Code

**Figure 5:**
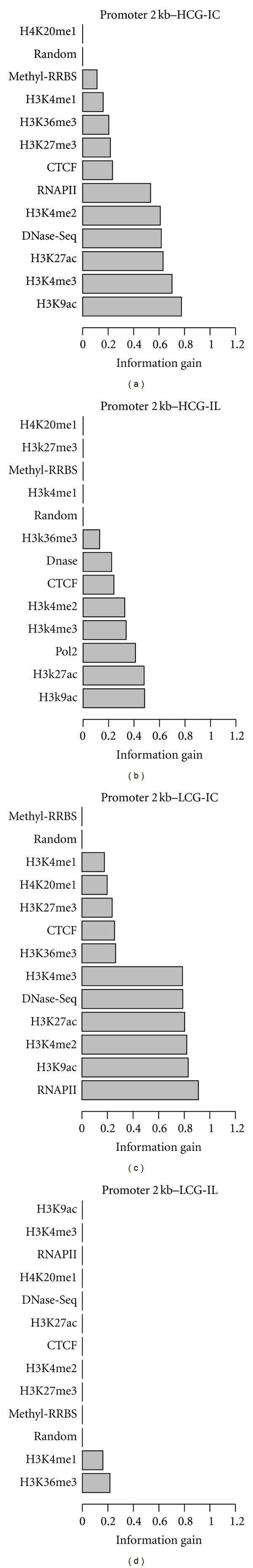
Information gain values measured for attributes in the 2 kb promoter region, comparing intron-less (IL) genes with intron-containing (IC) genes before filtering transcripts (Section 2). The compared sets were taken to be of the same size (105 transcript loci for HCGs and 84 transcript loci for LCGs).

A number of specific histone modifications have been related to the cotranscriptional splicing of introns [22, 38]. We, therefore, hypothesized that there should be relevant differences in the histone modifications between IC and IL loci. We thus compared the most informative attributes between intron-containing (IC) and intron-less (IL) loci (Figure 5 and Supplementary Figure 8). As there was many more IC than IL loci, we selected a subset of loci from IC of the same size as IL and compared the IG values for attributes related to fixed-length regions (Table 3). For HCG loci, although we found almost no differences when we ranked the attributes according to IG, there is an overall reduction of the IG values in IL genes. Strikingly, we found that for LCG loci the IG becomes very small for most of the attributes. For instance, in the promoter region, most of the attributes that are informative for LCG-IC loci do not contribute at all in LCG-IL; and H3K36me3, which is considered most relevant downstream of the TSS, and H3K4me1, which is not generally associated to an active TSS, become the most informative attribute for LCG-IL loci. Similarly, in the tail regions most of the attributes that are informative for LCG-IC loci do not contribute for LCG-IL loci, where the IG values are very low. In contrast, the tail region behaves more similarly for HCG-IC and HCG-IL, in terms of ranking and IG value. To further explore the differences in regulation between IC and IL genes, we calculated the profiles of reads for each mark in filtered transcript loci (Section 2). The profiles show large differences between expressed and nonexpressed IC genes (Supplementary Figure 2(A)) and confirm some of the already established locations of the marks relative to the loci. We also observe a striking difference of H3K36me3 read density around the pA in expressed versus nonexpressed IC genes, with a higher density around expressed genes. For IL genes, however, the signal is much weaker. This could be due to the fact that single exon genes tend to occur in families; hence, read mappability may be an issue. However, only 2% of IL genes overlap with low mappability regions, as classified at the UCSC Genome Browser. Nonetheless, we still observe differences between expressed and nonexpressed IL genes (Supplementary Figure 2(B)). For DNA methylation, we observe higher densities upstream of nonexpressed compared to expressed IL genes, consistent with earlier findings [7, 49]. However, we hardly see differences in DNA methylation for IC genes, which appear to be generally less methylated upstream of the TSS and more methylated downstream of the pA. The profile of pseudogenes, which have been excluded from the study of the expression code, are also shown in Supplementary Figure 2(C). Interestingly, although IC pseudogenes have much lower coverage of reads, they have similar profiles to the filtered IC genes, except for the transcription related signals: H3K4me1, RNAPII and H3K36me3, which show almost no signal, indicating nearly absent transcription.

## 4. Conclusions

A current challenge in epigenetics is how to extract biological knowledge from large volumes of data produced with new high-throughput technologies. Integrative tools and Machine-Learning (ML) algorithms are crucial to this aim. In this article, we have described a novel computational framework for the integration of high-throughput sequencing (HTS) epigenetic data that facilitates the generation and testing of quantitative models of gene regulation. Our methodology proposes a new way to relate epigenetic signals to expression using the comparison of the same locus between two conditions, instead of comparing loci to each other in a single condition, which can be affected by various biases. Three novel aspects of our methodology are that it (1) considers continuous values for the change in epigenetic signals, (2) it explores the enrichment of signals in multiple regions and (3) it can be applied to any HTS data type in two conditions.

We have shown the effectiveness of this methodology by building a predictive model of gene expression regulation based on epigenetic information for a pair of cell lines from the ENCODE project. The processed data used to build the models in this paper is available as a Biomart database at http://regulatorygenomics.upf.edu/group/pages/software/ Our quantitative models can predict whether a gene shows expression differences (up or down) or no difference between two cell lines. The relevant attributes and the accuracy for each model vary according to whether transcript loci have high CpG-content promoters (HCG) or not (LCG) and whether they contain introns (IC) or not (IL). These differences indicate that the histone signals are very heterogeneous and that regulation depends strongly on the actual structural properties of promoters and genes. Our analyses also indicate that there is high redundancy in the histone code, as different groups of attributes from different regions can explain a similar number of regulatory events.

Additionally, we have taken into account a fact largely overlooked in previous publications, which is that a considerable number of gene loci overlap with each other [50] at promoter and tail regions, or over their gene bodies, either on the same or on opposite strands. Accordingly, previous models of expression based on histone marks have this confounding effect, since the strand-less ChIP-Seq signal cannot be unambiguously associated to the regulation of a specific gene. Interestingly, when we removed these overlapping genes, the prediction accuracy improves considerably and the predictive model built from one pair of cell lines performs with high accuracy in a second pair of different cell lines. We conclude that removing these overlapping loci allows us to build a more general epigenetic code for expression regulation. This is further confirmed by our analysis of the information gain (IG), which shows that attributes can separate better the three regulatory classes after the overlapping loci are removed. Notably, this filtering does not change the ranking of IG values, hence although we improve the quantitative description of the histone code, the qualitative description does not change. The IG analysis confirms the role of some of the histone marks, like H3K9ac and H3K27ac, in the promoter and around the transcription start site in expression regulation as described before in the literature; and uncovers new regions, like the first intron for H3K36me3, the first exon for H3K4me3, and downstream of the polyadenylation site for H3K36me3, where changes in these marks associate strongly with expression regulation. The role of these marks in the first exon and intron indicates a general role in the coupling between splicing and transcription, as recently shown in the literature. In this direction, we also explored the patterns of epigenetic changes between intron-containing (IC) and intron-less (IL) loci and found that IC loci contain more epigenetic information and can therefore be better characterised. These differences are more remarkable between high (HCG) and low CpG promoters (LCG), which suggest that the type of promoter might influence the epigenetic changes that take place in cotranscriptional splicing [22]. Alternatively, this could indicate that these loci have a distinct mode of regulation, possibly by other marks that have not been considered in this study. 

The epigenetic signals analysed in this study provide a strong prediction power for expression regulation. However, there is still a proportion of genes for which their change in expression cannot be explained by the changes of the studied signals. In any case, the associations found do not necessarily imply causality or a direct functional effect, as the effect of a given histone mark may be context dependent and may occur through the action of other factors. Nonetheless, the models described reflect the complex network of gene regulation and provide some of the generic features of this network. Our methodology provides an effective way to integrate the continuous changes in epigenetic signals between different conditions. Applying this approach to datasets with more histone modifications and transcription factors will help expanding and characterizing further this complex regulatory network. In particular, the application of our approach to different developmental stages, disease states, or treatments, will help uncovering the epigenetic mechanisms responsible for cellular differentiation and carcinogenesis.

## Supplementary Material

The Supplementary Material contains plots related to all the attributes, regions and gene-sets, described in the text, providing exhaustive information and complementing the ones presented in the main text. In particular, it includes the read profiles of the marks for the various gene sets, pair-wise correlations of marks for all regions and gene sets, z-score distributions for informative and non-informative attributes not included in the main text, the attribute Information Gain values for all regions before and after removing overlapping genes, Information Gain value comparison between intron-less and introncontaining genes for all considered regions, accuracy evaluation of the predictions on the intron-less genes, the exhaustive list of informative features obtained from the different training sets, the list of most informative attributes per genic region and the accuracy achieved using those, and the Pyicos command lines used to perform the analyses.

## Figures and Tables

**Table 1:** Each of the four sets of transcript loci considered in our analysis. The numbers correspond to the loci before (all) or after (filtered) eliminating overlapping loci (Section 2). From each set, we considered up-, down-, or nonregulated transcript loci, each corresponding to 1/3 of the indicated numbers.

Transcript-loci set	Description	Pair 1-all	Pair 1-filtered	Pair 2-all	Pair 2-filtered
HCG IC	High CG promoter and intron-containing	6510	1959	2964	792
HCG IL	High CG promoter and intron-less	105	27	24	12
LCG IC	Low CG promoter and intron-containing	6705	1767	1980	585
LCG IL	Low CG promoter and intron-less	84	30	15	15

**Table 2:** ENCODE data sets and cell lines used for analysis: ChIP-Seq data for RNA Polymerase II (RNAPII), CTCF and various Histone marks, data for DNase I hypersensitive sites (DNase-Seq), methylation data from reduced representation bisulfite sequencing (methyl-RRBS) and sequencing of long polyA+ whole cell RNA (RNA-Seq). For HMEC and HSMM cells RNAPII ChIP-Seq data was not available at the time of our analyses. Datasets were generated at the Broad Institute (BROAD), Cold Spring Harbor Laboratory (CSHL), University of Washington (UW), University of Texas at Austin (UT-A), and Hudson Alpha (HA).

Factor/mark	Pair 1		Pair 2	
	Cell lines		Cell lines	
	K562	GM12878	HSMM	HMEC
CTCF	BROAD	BROAD	BROAD	BROAD
H3K27ac	BROAD	BROAD	BROAD	BROAD
H3K27me3	BROAD	BROAD	BROAD	BROAD
H3K36me3	BROAD	BROAD	BROAD	BROAD
H3K4me1	BROAD	BROAD	BROAD	BROAD
H3K4me2	BROAD	BROAD	BROAD	BROAD
H3K4me3	BROAD	BROAD	BROAD	BROAD
H3K9ac	BROAD	BROAD	BROAD	BROAD
H4K20me1	BROAD	BROAD	BROAD	BROAD
RNAPII	UT-A	UT-A	—	—
DNase-Seq	UW	UW	UW	UW
Methyl-RRBS	HA	HA	HA	HA
RNA-Seq	CSHL	CSHL	CSHL	CSHL

**Table 3:** Regions considered per transcript locus for the calculation of the different attributes. We defined the 13 regions based on the gene annotations from Gencode version 7 (Ensembl 62).

Type	Region	Description
	Promoter 2 kb	Region starting 2 kb upstream of the transcription start site (TSS) and ending 1 bp before the TSS;
	Promoter 5 kb	Region starting 5 kb upstream of the TSS and ending 1 bp before the TSS;
Fixed-length regions	TSS ± 2 kb	Region starting 2 kb upstream of the TSS and ending 2 kb downstream
	TSS ± 5 kb	Region starting 5 kb upstream of the TSS and ending 5 kb downstream
	pA ± 2 kb	Region starting 2 kb upstream of the pA and ending 2 kb downstream
	Tail	Region starting 1 bp after the pA and ending 2 kb downstream

	First exon	Region corresponding to the first exon of the transcript locus
	First intron	Region corresponding to the first intron of the transcript locus
	GB	Gene body, that is, region between the TSS and the poly-adenylation site (pA) of an annotated transcript locus
Variable-length regions	GB3′ss	Region between the first 3′ splice-site and the pA of an annotated transcript locus
	GB ± 1 kb	Gene body with additional 1 kb stretches up- and downstream
	GB ± 5 kb	Gene body with additional 5 kb stretches up- and downstream
	GB + 5 kb	Gene body with an additional 5kb stretch downstream of the pA

**Table:** (a) Before filtering

Attributes	HCG-IC				LCG-IC			
	Up	Dw	Nr	Average	Up	Dw	Nr	Average
P1 (with RNAPII)	0.8	0.79	0.74	0.78	0.82	0.87	0.78	0.83
P1	0.79	0.79	0.74	0.77	0.83	0.86	0.76	0.82
P1 (CFS)	0.8	0.79	0.74	0.78	0.82	0.86	0.76	0.81

P2	0.85	0.83	0.81	0.83	0.9	0.88	0.83	0.87
P2 (CFS-P1)	0.85	0.83	0.8	0.83	0.9	0.88	0.83	0.87
P1-on-P2	0.83	0.77	0.63	0.74	0.88	0.83	0.71	0.81
P1(CFS)-on-P2	0.83	0.8	0.57	0.73	0.88	0.84	0.74	0.82

**Table:** (b) After filtering

Attributes	HCG-IC				LCG-IC			
	Up	Dw	Nr	Average	Up	Dw	Nr	Average
P1 (with RNAPII)	0.79	0.84	0.76	0.8	0.85	0.9	0.81	0.86
P1	0.79	0.82	0.75	0.79	0.86	0.89	0.76	0.84
P1 (CFS)	0.79	0.81	0.73	0.78	0.84	0.9	0.77	0.84

P2	0.89	0.88	0.85	0.87	0.92	0.91	0.85	0.89
P2 (CFS-P1)	0.87	0.87	0.84	0.86	0.92	0.92	0.86	0.9
P1-on-P2	0.89	0.87	0.7	0.82	0.92	0.89	0.79	0.87
P1(CFS)-on-P2	0.85	0.82	0.68	0.78	0.91	0.89	0.81	0.87

**Table 5:** Correctly classified instances in each transcript subset. Sets are filtered to avoid overlapping gene bodies, promoters or tails from transcript loci from different genes in the same or opposite strands (Section 2). Attribute selection has been applied to each pair: P1 (CFS) and P2 (CFS), for each of the subsets of intron-containing loci, high (HCG) or low (LCG) CG content promoter. The attribute sets correspond to the ones from Table 4(b): P1 (CFS) denotes the model for P1, where the attributes used are those that have a score 80 or higher (maximum 100) using the CFS attribute selection method. P2 (CFS-P1) indicates that the model was trained using the data from P2 but the attributes selected using CFS on P1. P1 (CFS)-on-P2 indicates that the model was trained with pair P1 with only selected attributes and tested on pair P2.

Attributes	Transcript loci set	Instances in total	Correctly classified instances
P1 (CSF)	LCG-IC	1767	1185 (67.06%)
	HCG-IC	1959	1182 (60.34%)
P2 (CSF-P1)	LCG-IC	585	454 (77.60%)
	HCG-IC	792	577 (72.85%)
P1 (CSF)-on-P2	LCG-IC	585	410 (70.09%)
	HCG-IC	792	445 (56.19%)

## References

[1] Kornberg RD, Thomas JO (1974). Chromatin structure: oligomers of the histones. *Science*.

[2] Li B, Carey M, Workman JL (2007). The Role of chromatin during transcription. *Cell*.

[3] Mellor J, Dudek P, Clynes D (2008). A glimpse into the epigenetic landscape of gene regulation. *Current Opinion in Genetics and Development*.

[4] Pokholok DK, Harbison CT, Levine S (2005). Genome-wide map of nucleosome acetylation and methylation in yeast. *Cell*.

[5] Joshi AA, Struhl K (2005). Eaf3 chromodomain interaction with methylated H3-K36 links histone deacetylation to pol II elongation. *Molecular Cell*.

[6] Lister R, Pelizzola M, Kida YS (2011). Hotspots of aberrant epigenomic reprogramming in human induced pluripotent stem cells. *Nature*.

[7] Ellis L, Atadja PW, Johnstone RW (2009). Epigenetics in cancer: targeting chromatin modifications. *Molecular Cancer Therapeutics*.

[8] Kulis M, Esteller M (2010). DNA methylation and cancer. *Advances in Genetics*.

[9] Jenuwein T, Allis CD (2001). Translating the histone code. *Science*.

[10] Barski A, Cuddapah S, Cui K (2007). High-resolution profiling of histone methylations in the human genome. *Cell*.

[11] Yu H, Zhu S, Zhou B, Xue H, Han JDJ (2008). Inferring causal relationships among different histone modifications and gene expression. *Genome Research*.

[12] Hon G, Ren B, Wang W (2008). ChromaSig: a probabilistic approach to finding common chromatin signatures in the human genome. *PLoS Computational Biology*.

[13] Van Steensel B, Braunschweig U, Filion GJ, Chen M, Van Bemmel JG, Ideker T (2010). Bayesian network analysis of targeting interactions in chromatin. *Genome Research*.

[14] Karlić R, Chung HR, Lasserre J, Vlahoviček K, Vingron M (2010). Histone modification levels are predictive for gene expression. *Proceedings of the National Academy of Sciences of the United States of America*.

[15] Ernst J, Kellis M (2010). Discovery and characterization of chromatin states for systematic annotation of the human genome. *Nature Biotechnology*.

[16] Cheng C, Gerstein M (2012). Modeling the relative relationship of transcription factor binding and histone modifications to gene expression levels in mouse embryonic stem cells. *Nucleic Acids Research*.

[17] Ernst J, Kheradpour P, Mikkelsen TS (2011). Mapping and analysis of chromatin state dynamics in nine human cell types. *Nature*.

[18] Jenuwein T, Allis CD (2001). Translating the histone code. *Science*.

[19] Strahl BD, Allis CD (2000). The language of covalent histone modifications. *Nature*.

[20] Robertson G, Hirst M, Bainbridge M (2007). Genome-wide profiles of STAT1 DNA association using chromatin immunoprecipitation and massively parallel sequencing. *Nature Methods*.

[21] Mortazavi A, Williams BA, McCue K, Schaeffer L, Wold B (2008). Mapping and quantifying mammalian transcriptomes by RNA-Seq. *Nature Methods*.

[22] de Almeida SF, Grosso AR, Koch F (2011). Splicing enhances recruitment of methyltransferase HYPB/Setd2 and methylation of histone H3 Lys36. *Nature Structural and Molecular Biology*.

[23] Cheung M-S, Down TA, Latorre I, Ahringer J (2011). Systematic bias in high-throughput sequencing data and its correction by BEADS. *Nucleic Acids Research*.

[24] Teytelman L, Özaydin B, Zill O (2009). Impact of chromatin structures on DNA processing for genomic analyses. *PLoS ONE*.

[25] Auerbach RK, Euskirchen G, Rozowsky J (2009). Mapping accessible chromatin regions using Sono-Seq. *Proceedings of the National Academy of Sciences of the United States of America*.

[26] Park PJ (2009). ChIP-seq: advantages and challenges of a maturing technology. *Nature Reviews Genetics*.

[27] Rozowsky J, Euskirchen G, Auerbach RK (2009). PeakSeq enables systematic scoring of ChIP-seq experiments relative to controls. *Nature Biotechnology*.

[28] Dohm JC, Lottaz C, Borodina T, Himmelbauer H (2008). Substantial biases in ultra-short read data sets from high-throughput DNA sequencing. *Nucleic Acids Researchearch*.

[29] Myers RM, Stamatoyannopoulos J, Snyder M, Dunham I, Hardison RC, Bernstein BE (2011). A user’s guide to the encyclopedia of DNA elements (ENCODE). *PLoS Biology*.

[30] Boyle AP, Davis S, Shulha HP (2008). High-resolution mapping and characterization of open chromatin across the genome. *Cell*.

[31] Meissner A, Gnirke A, Bell GW, Ramsahoye B, Lander ES, Jaenisch R (2005). Reduced representation bisulfite sequencing for comparative high-resolution DNA methylation analysis. *Nucleic Acids Research*.

[32] Flicek P, Amode MR, Barrell D, Beal K, Brent S, Carvalho-Silva D (2012). Ensembl 2012. *Nucleic Acids Research*.

[33] Dreszer TR, Karolchik D, Zweig AS, Hinrichs AS, Raney BJ, Kuhn RM (2012). The UCSC Genome Browser database: extensions and updates 2011. *Nucleic Acids Research*.

[34] Kasprzyk A (2011). BioMart: driving a paradigm change in biological data management. *Database (Oxford)*.

[35] Frank E, Hall M, Trigg L, Holmes G, Witten IH (2004). Data mining in bioinformatics using Weka. *Bioinformatics*.

[36] Hoang SA, Xu X, Bekiranov S (2011). Quantification of histone modification ChIP-seq enrichment for data mining and machine learning applications. *BMC Research Notes*.

[37] Huff JT, Plocik AM, Guthrie C, Yamamoto KR (2010). Reciprocal intronic and exonic histone modification regions in humans. *Nature Structural and Molecular Biology*.

[38] Sims RJ, Millhouse S, Chen CF (2007). Recognition of Trimethylated Histone H3 Lysine 4 Facilitates the Recruitment of Transcription Postinitiation Factors and Pre-mRNA Splicing. *Molecular Cell*.

[39] Carninci P, Sandelin A, Lenhard B (2006). Genome-wide analysis of mammalian promoter architecture and evolution. *Nature Genetics*.

[40] Saxonov S, Berg P, Brutlag DL (2006). A genome-wide analysis of CpG dinucleotides in the human genome distinguishes two distinct classes of promoters. *Proceedings of the National Academy of Sciences of the United States of America*.

[41] Valen E, Sandelin A (2011). Genomic and chromatin signals underlying transcription start-site selection. *Trends in Genetics*.

[42] Breiman L (2001). Random forests. *Machine Learning*.

[43] Schug J, Schuller WP, Kappen C, Salbaum JM, Bucan M, Stoeckert CJ (2005). Promoter features related to tissue specificity as measured by Shannon entropy. *Genome Biology*.

[44] Zhang Z, Zhang MQ (2011). Histone modification profiles are predictive for tissue/cell-type specific expression of both protein-coding and microRNA genes. *BMC Bioinformatics*.

[45] Hall M (1999). *Correlation-based feature selection for machine learning [Ph.D. thesis]*.

[46] Mitchell T (1997). *Machine Learning*.

[47] Wang Z, Zang C, Rosenfeld JA (2008). Combinatorial patterns of histone acetylations and methylations in the human genome. *Nature Genetics*.

[48] Sims JK, Houston SI, Magazinnik T, Rice JC (2006). A trans-tail histone code defined by monomethylated H4 Lys-20 and H3 Lys-9 demarcates distinct regions of silent chromatin. *The Journal of Biological Chemistry*.

[49] Bird A (1992). The essentials of DNA methylation. *Cell*.

[50] Katayama S, Tomaru Y, Kasukawa T (2005). Molecular biology: antisense transcription in the mammalian transcriptome. *Science*.

