# Supplementary material for: Predictive Models of Gene Regulation from High-Throughput Epigenomics Data
Source: Comp Funct Genomics. 2012 Aug 13;2012:284786. doi: 10.1155/2012/284786 (PMC3424690; doi:10.1155/2012/284786)
Supplement: Supplementary file 1 — The Supplementary Material contains plots related to all the attributes, regions and gene-sets, described in the text, providing exhaustive information and complementing the ones presented in the main text. In particular, it includes the read profiles of the marks for the various gene sets, pair-wise correlations of marks for all regions and gene sets, z-score distributions for informative and non-informative attributes not included in the main text, the attribute Information Gain values for all regions before and after removing overlapping genes, Information Gain value comparison between intron-less and introncontaining genes for all considered regions, accuracy evaluation of the predictions on the intron-less genes, the exhaustive list of informative features obtained from the different training sets, the list of most informative attributes per genic region and the accuracy achieved using those, and the Pyicos command lines used to perform the analyses. [file 284786.f1.pdf]

Supplementary File

Predictive models of gene regulation from high-throughput epigenomics data

Sonja Althammer[1], Amadís Pagès[1], Eduardo Eyras[1,2,*]

[1]Computational Genomics, Universitat Pompeu Fabra, Dr. Aiguader 88, E08003 Barcelona, Spain

[2]Institució Catalana de Recerca i Estudis Avançats (ICREA), Passeig Lluís Companys 23, E08010 Barcelona, Spain

[*]To whom the correspondence should be addressed: eduardo.eyras@upf.edu

Keywords: chromatin, machine learning, regulation, histone code, ChIP-Seq, high-throughput sequencing, differential expression

Supplementary Figures

Supplementary Figure 1: Different configurations of overlapping transcripts from different genes. We removed pairs of different genes that had transcripts overlapping in these configurations, as they would make ambiguous the assignment of the chromatin signal to the right transcript locus. We show in the figure the various types of overlap considered. Clockwise from the top left: overlapping 2kb tail regions (tail_2kb) in opposite strands, overlapping transcripts from different genes in the same strand, tail and promoter (pr_2kb) overlapping in opposite strands, promoters (pr_2kb) overlapping in opposite strands, and transcript bodies overlapping in opposite strands. Excluding these genes cases results in general into a more accurate predictive model.

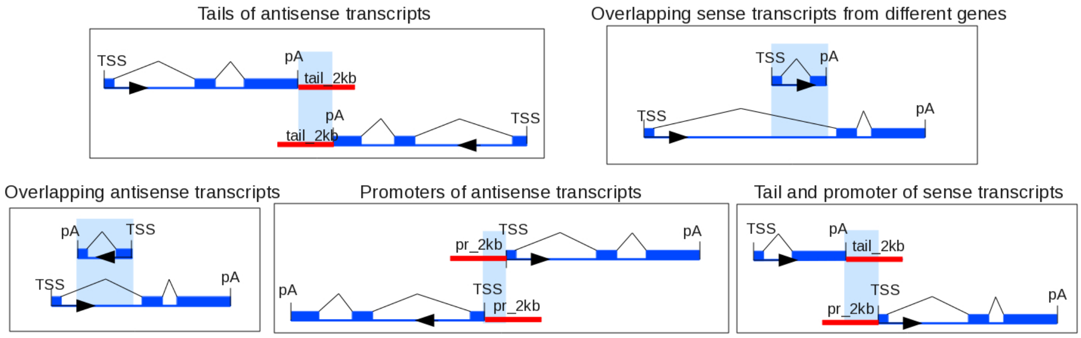

Supplementary Figure 2:

A) Profiles of epigenetic marks around the genebodies of expressed and non-expressed IC and IL genes. See methods.

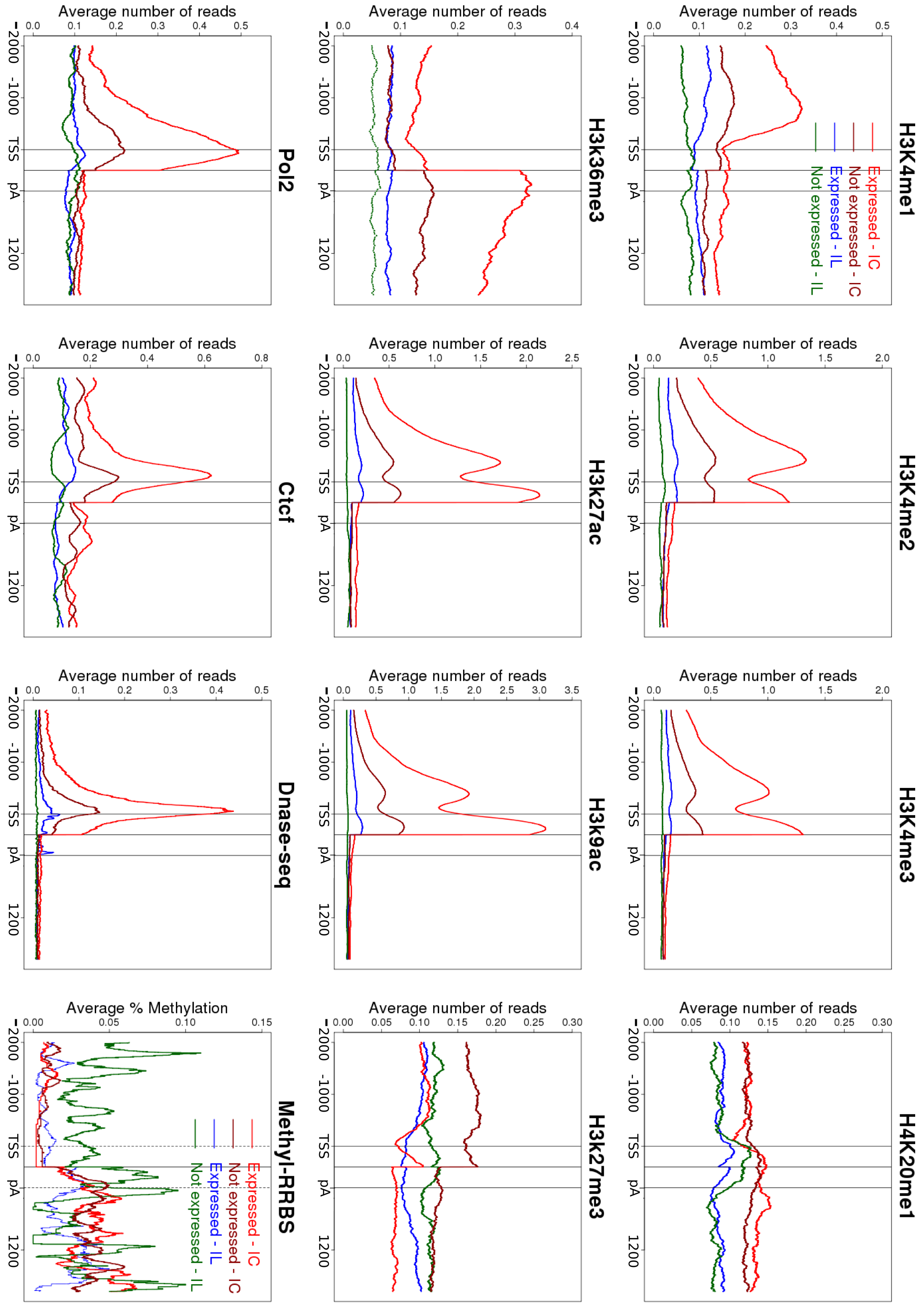

B) Profiles of epigenetic marks around the genebodies of expressed and non-expressed IL genes. See methods

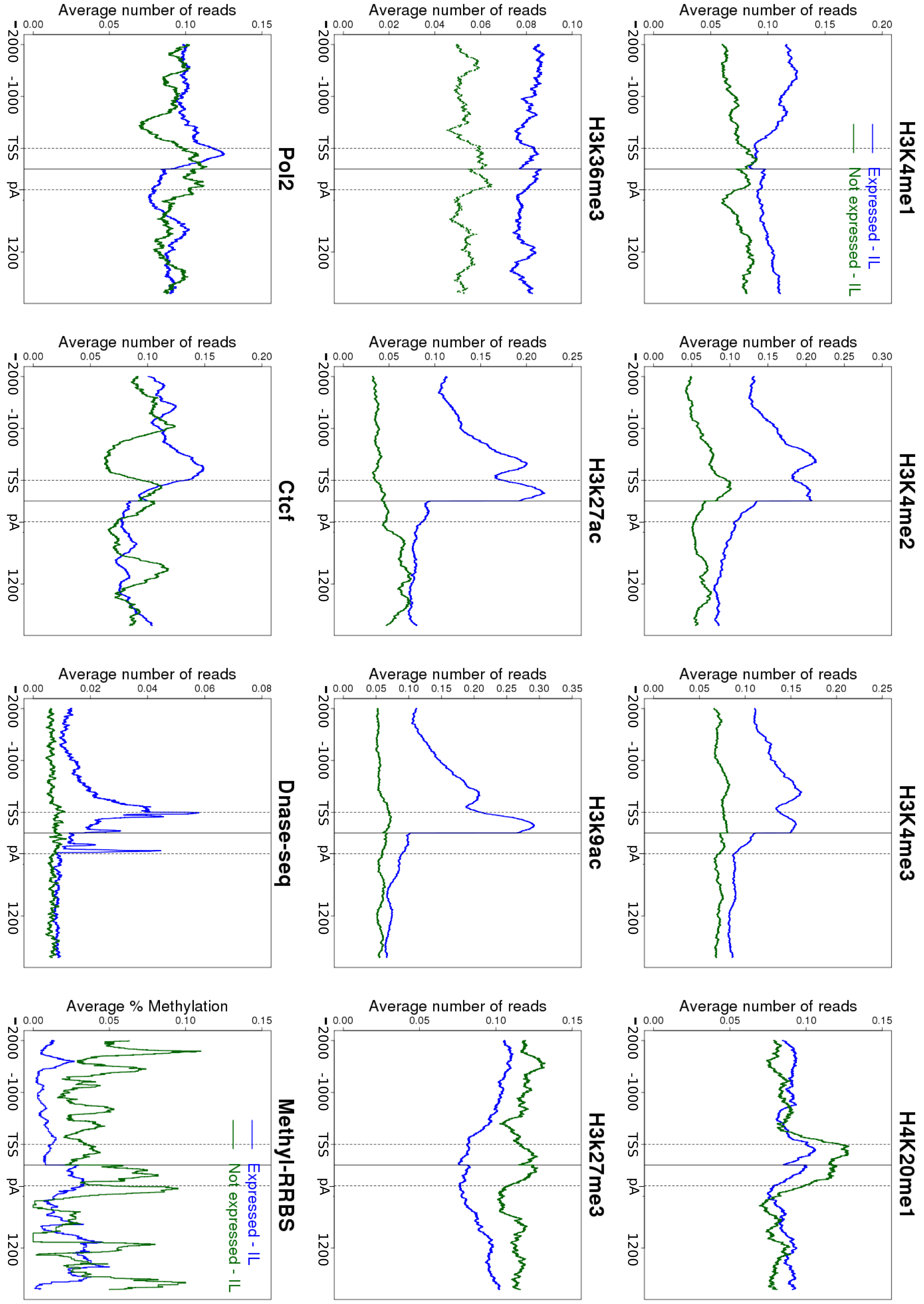

C) Profiles of epigenetic marks around the genebodies of expressed and non-expressed IC and IL pseudogenes. See methods.

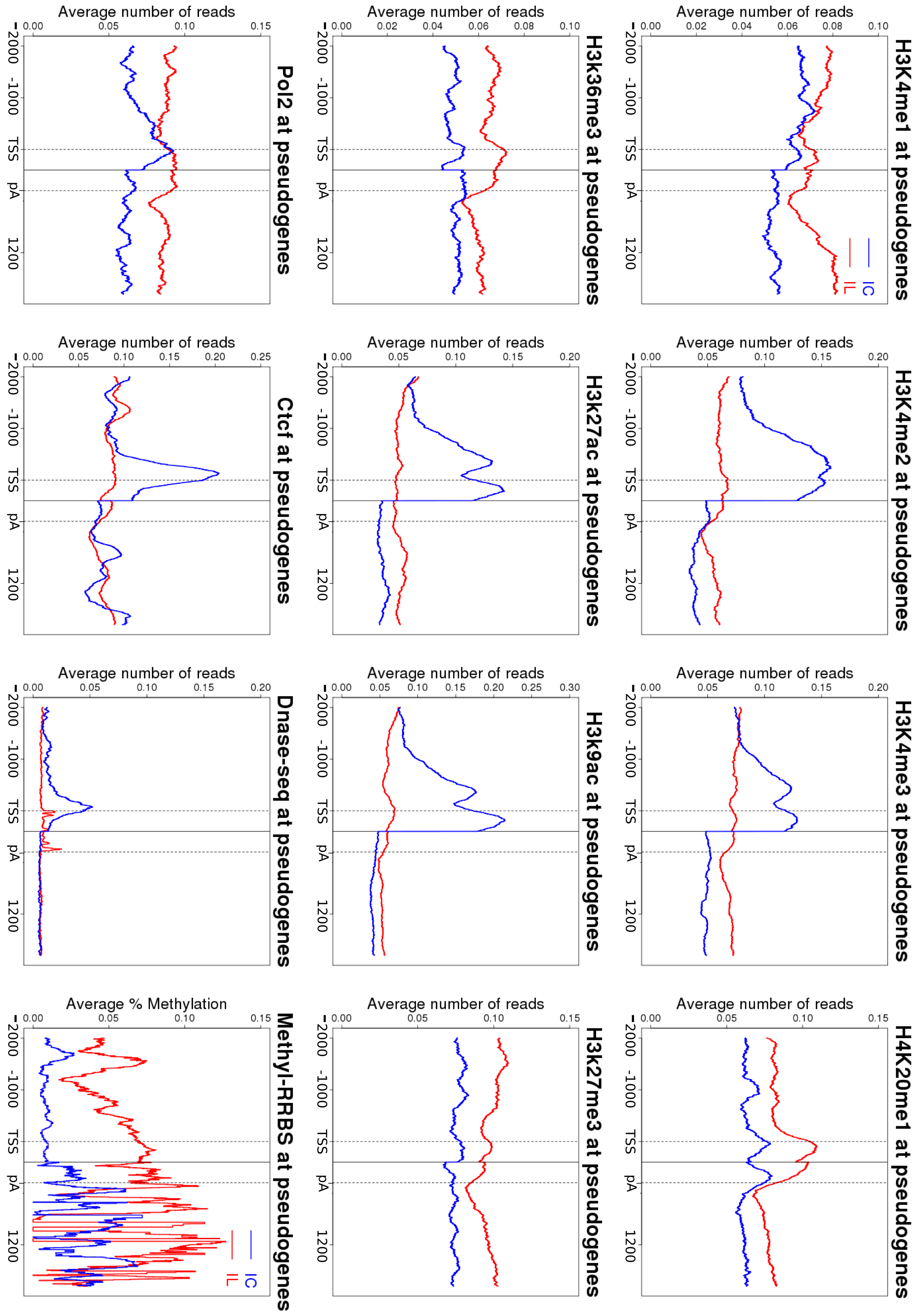

Supplementary Figure 3: Pairwise correlations of marks and expression changes at regulated loci. Heatmaps are shown for regulated genes from the filtered intron-containing (IC) sets for high (HCG) and low (LCG) CpG promoters. The color represents the value of the Pearson correlation coefficient between the z-scores for every pair of attributes. For expression (RNA-Seq), the z-scores of the Up and Dw transcript loci were used to calculate the correlation. For all heatmaps, each color always represents the same value, which may span a different range of values in each heatmap.

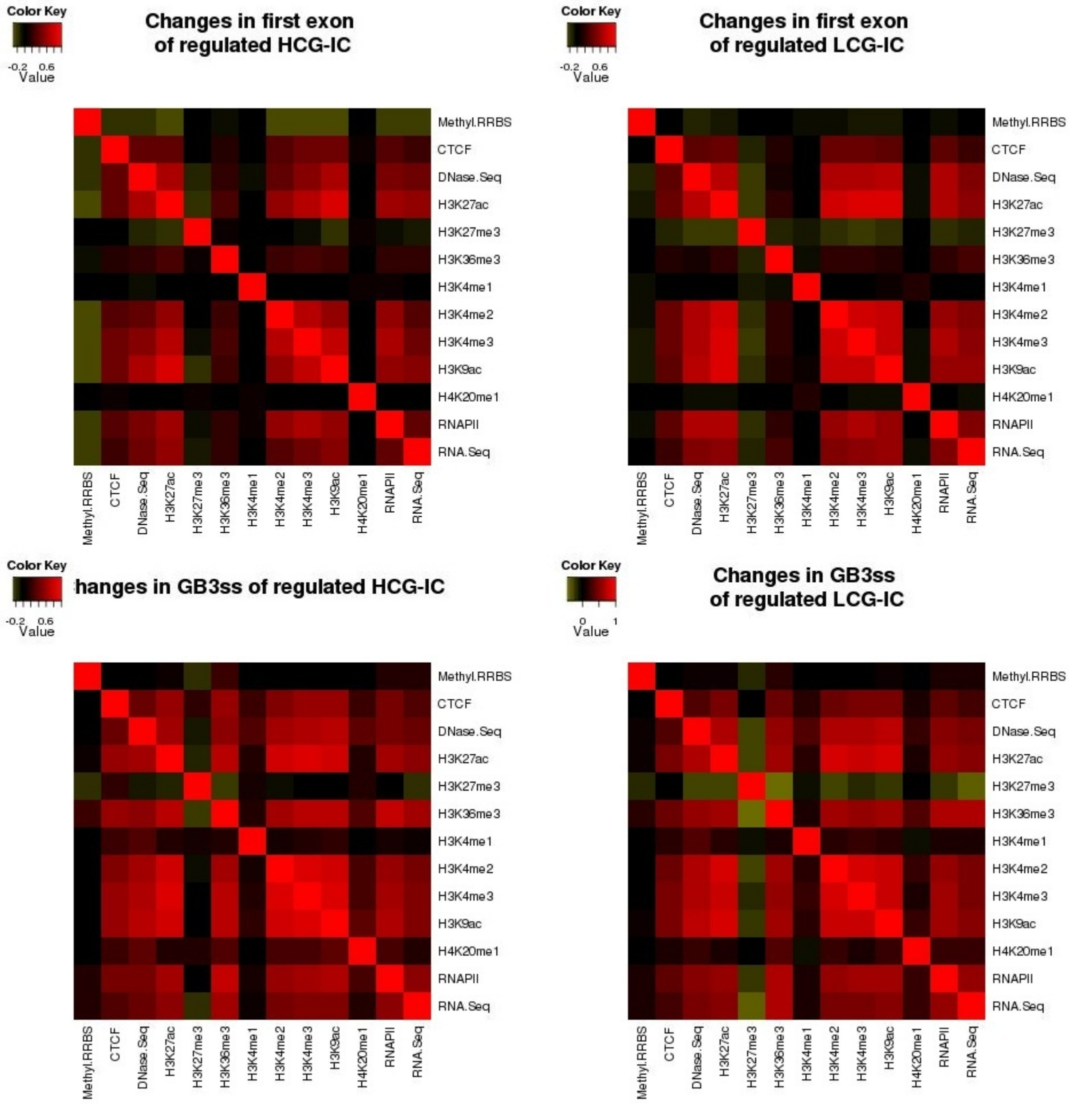

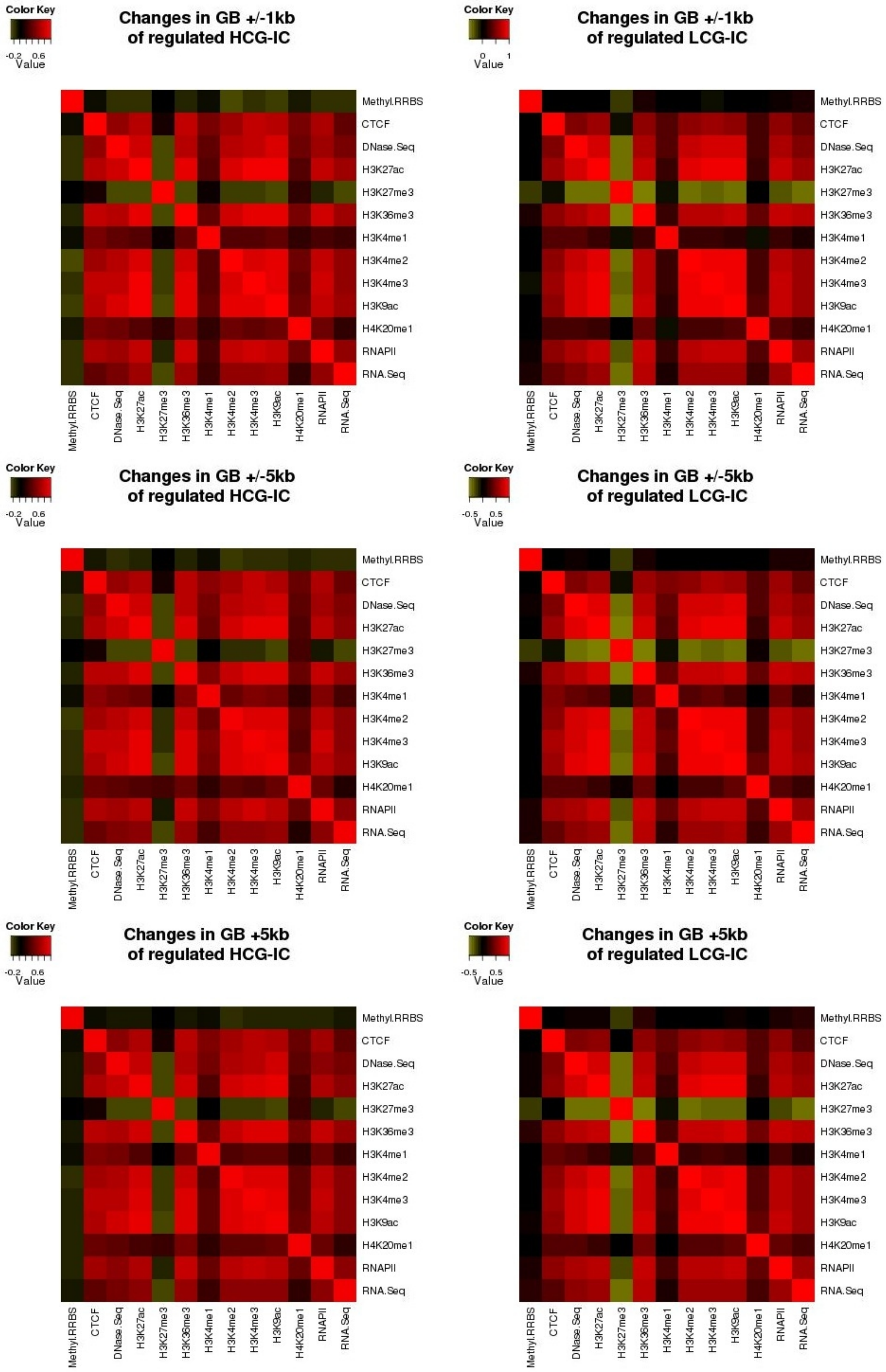
Color Key
-0.2 0.6
Value
Changes in GB +/-1kb of regulated HCG-IC
Color Key
0 1
Value
Changes in GB +/-1kb of regulated LCG-IC
Color Key
-0.2 0.6
Value
Changes in GB +/-5kb of regulated HCG-IC
Color Key
-0.5 0.5
Value
Changes in GB +/-5kb of regulated LCG-IC
Color Key
-0.2 0.6
Value
Changes in GB +5kb of regulated HCG-IC
Color Key
-0.5 0.5
Value
Changes in GB +5kb of regulated LCG-IC
Methyl.RRBS
CTCF
DNase.Seq
H3K27ac
H3K27me3
H3K36me3
H3K4me1
H3K4me2
H3K4me3
H3K9ac
H4K20me1
RNAPII
RNA.Seq

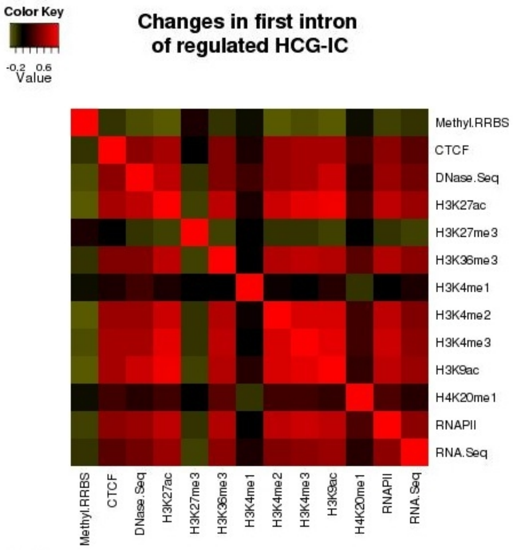
Color Key
-0.2 0.6
Value
Changes in first intron
of regulated HCG-IC
Methyl.RRBS
CTCF
DNase.Seq
H3K27ac
H3K27me3
H3K36me3
H3K4me1
H3K4me2
H3K4me3
H3K9ac
H4K20me1
RNAPII
RNA.Seq

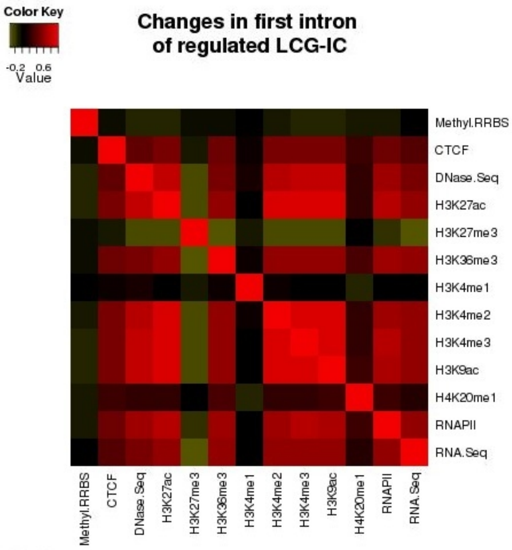
Color Key
-0.2 0.6
Value
Changes in first intron
of regulated LCG-IC
Methyl.RRBS
CTCF
DNase.Seq
H3K27ac
H3K27me3
H3K36me3
H3K4me1
H3K4me2
H3K4me3
H3K9ac
H4K20me1
RNAPII
RNA.Seq

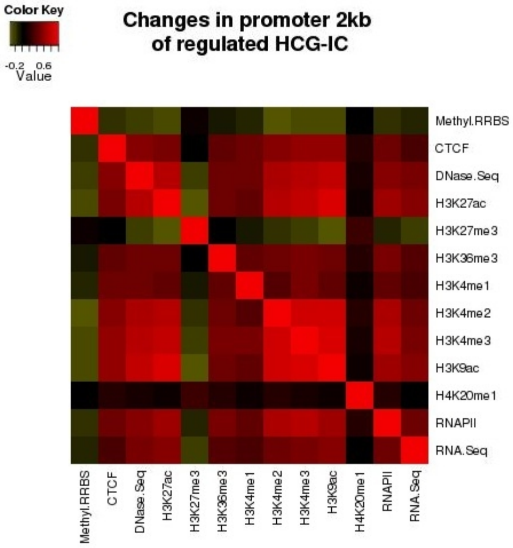
Color Key
-0.2 0.6
Value
Changes in promoter 2kb
of regulated HCG-IC
Methyl.RRBS
CTCF
DNase.Seq
H3K27ac
H3K27me3
H3K36me3
H3K4me1
H3K4me2
H3K4me3
H3K9ac
H4K20me1
RNAPII
RNA.Seq

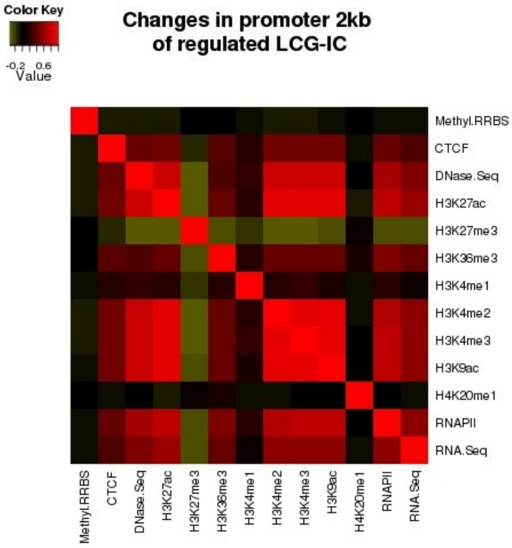
Color Key
-0.2 0.6
Value
Changes in promoter 2kb
of regulated LCG-IC
Methyl.RRBS
CTCF
DNase.Seq
H3K27ac
H3K27me3
H3K36me3
H3K4me1
H3K4me2
H3K4me3
H3K9ac
H4K20me1
RNAPII
RNA.Seq

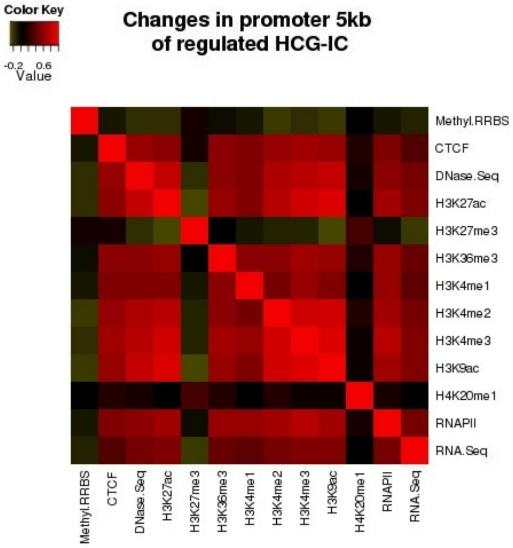
Color Key
-0.2 0.6
Value
Changes in promoter 5kb
of regulated HCG-IC
Methyl.RRBS
CTCF
DNase.Seq
H3K27ac
H3K27me3
H3K36me3
H3K4me1
H3K4me2
H3K4me3
H3K9ac
H4K20me1
RNAPII
RNA.Seq

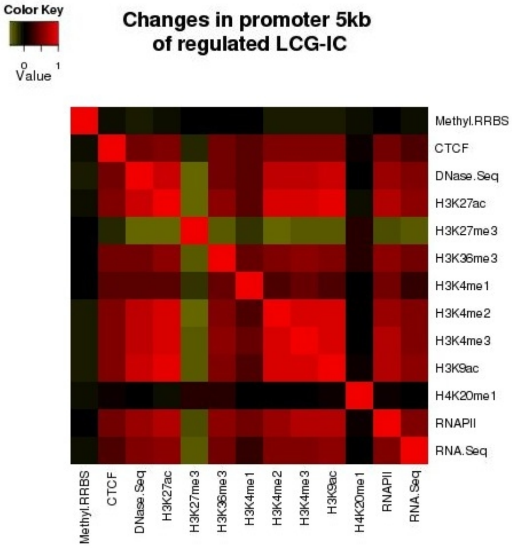
Color Key
0 1
Value
Changes in promoter 5kb
of regulated LCG-IC
Methyl.RRBS
CTCF
DNase.Seq
H3K27ac
H3K27me3
H3K36me3
H3K4me1
H3K4me2
H3K4me3
H3K9ac
H4K20me1
RNAPII
RNA.Seq

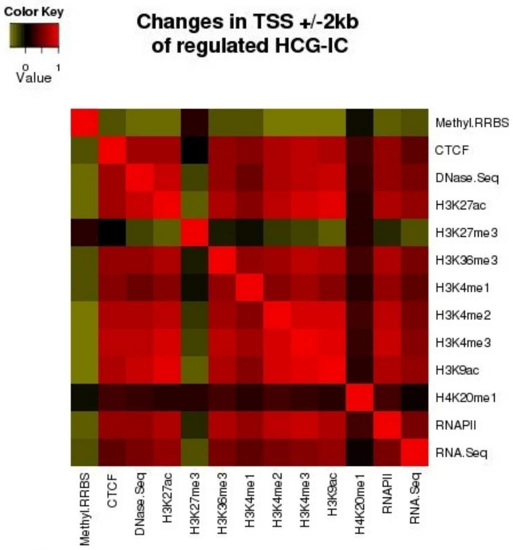
Color Key
0
1
Value
Changes in TSS +/-2kb
of regulated HCG-IC
Methyl.RRBS
CTCF
DNase.Seq
H3K27ac
H3K27me3
H3K36me3
H3K4me1
H3K4me2
H3K4me3
H3K9ac
H4K20me1
RNAPII
RNA.Seq

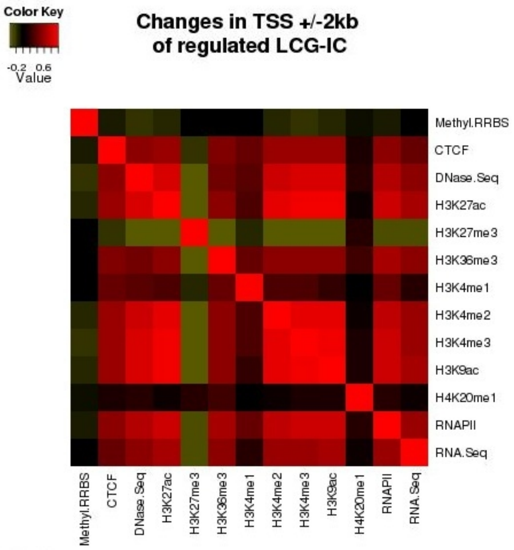
Color Key
-0.2
0.6
Value
Changes in TSS +/-2kb
of regulated LCG-IC
Methyl.RRBS
CTCF
DNase.Seq
H3K27ac
H3K27me3
H3K36me3
H3K4me1
H3K4me2
H3K4me3
H3K9ac
H4K20me1
RNAPII
RNA.Seq

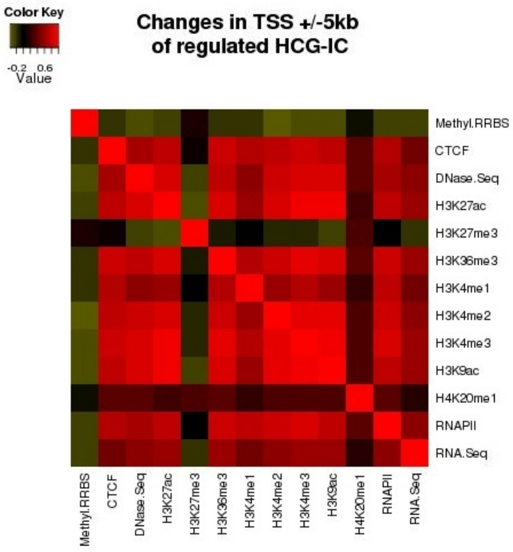
Color Key
-0.2
0.6
Value
Changes in TSS +/-5kb
of regulated HCG-IC
Methyl.RRBS
CTCF
DNase.Seq
H3K27ac
H3K27me3
H3K36me3
H3K4me1
H3K4me2
H3K4me3
H3K9ac
H4K20me1
RNAPII
RNA.Seq

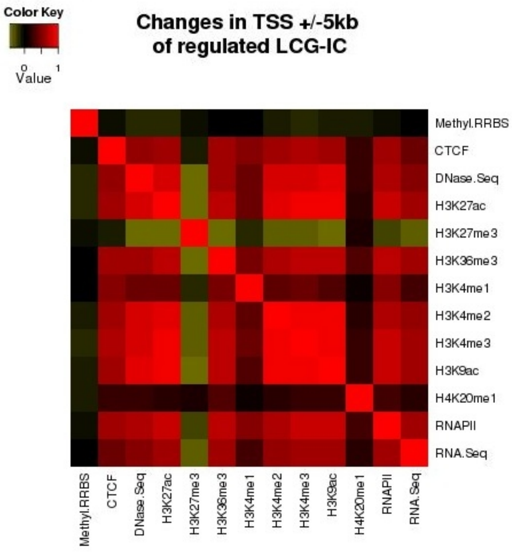
Color Key
0
1
Value
Changes in TSS +/-5kb
of regulated LCG-IC
Methyl.RRBS
CTCF
DNase.Seq
H3K27ac
H3K27me3
H3K36me3
H3K4me1
H3K4me2
H3K4me3
H3K9ac
H4K20me1
RNAPII
RNA.Seq

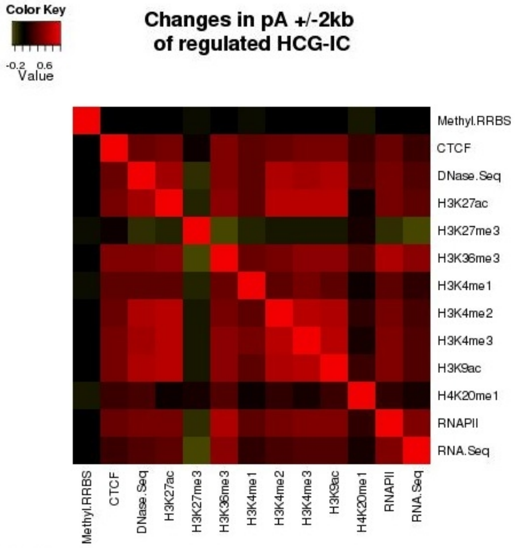
Color Key
-0.2 0.6
Value
Changes in pA +/-2kb of regulated HCG-IC
Methyl.RRBS
CTCF
DNase.Seq
H3K27ac
H3K27me3
H3K36me3
H3K4me1
H3K4me2
H3K4me3
H3K9ac
H4K20me1
RNAPII
RNA.Seq

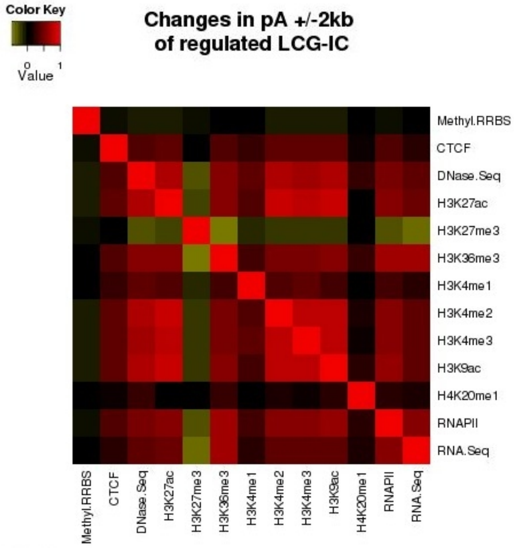
Color Key
0 1
Value
Changes in pA +/-2kb of regulated LCG-IC
Methyl.RRBS
CTCF
DNase.Seq
H3K27ac
H3K27me3
H3K36me3
H3K4me1
H3K4me2
H3K4me3
H3K9ac
H4K20me1
RNAPII
RNA.Seq

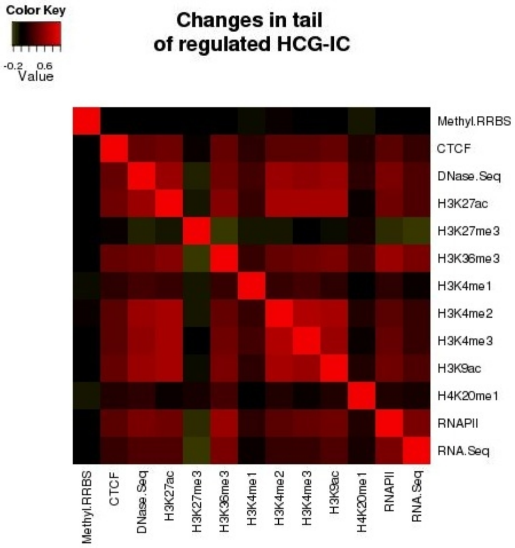
Color Key
-0.2 0.6
Value
Changes in tail of regulated HCG-IC
Methyl.RRBS
CTCF
DNase.Seq
H3K27ac
H3K27me3
H3K36me3
H3K4me1
H3K4me2
H3K4me3
H3K9ac
H4K20me1
RNAPII
RNA.Seq

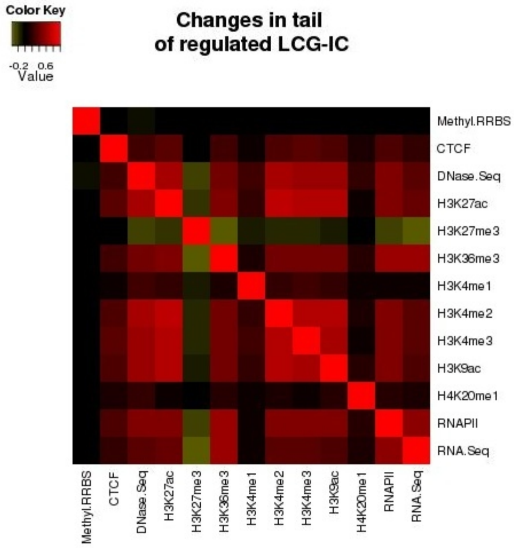
Color Key
-0.2 0.6
Value
Changes in tail of regulated LCG-IC
Methyl.RRBS
CTCF
DNase.Seq
H3K27ac
H3K27me3
H3K36me3
H3K4me1
H3K4me2
H3K4me3
H3K9ac
H4K20me1
RNAPII
RNA.Seq

Supplementary Figure 4: Distribution of z-scores of some of the best separating attributes for False Positives and True Positives. False Positive (FP) instances (upper panels) show a similar distribution of z-scores centred around zero for all three groups of up (Up), down (Dw) and non-regulated (Nr) genes for two of the best separating attributes. However, True Positive (TP) instances (lower panels) show a clear separation of z-scores between Up, Dw and Nr instances. The prediction potential of these attributes is reflected by z-score distributions for Up, Dw and Nr genes.

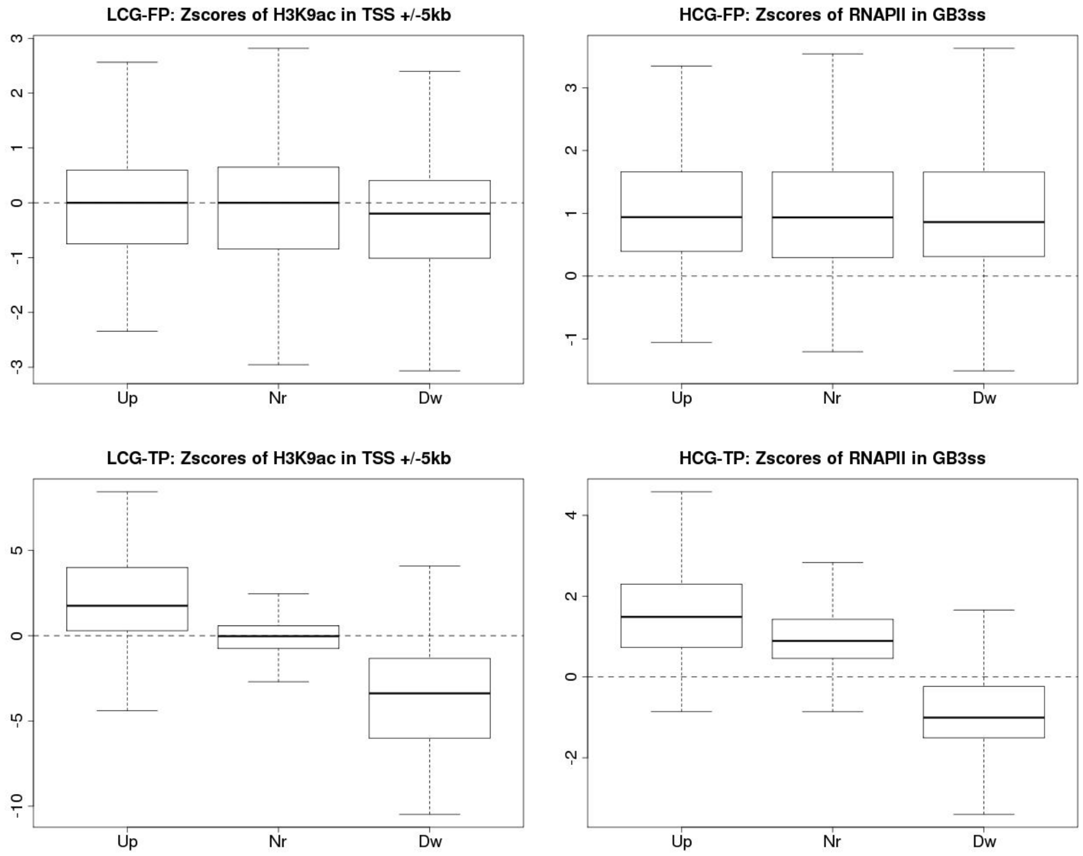

Supplementary Figure 5: Information Gain before and after removing ambiguous signals (pair1). The panels show the ranking of the attributes according to Information Gain before (left) and after (right) removing overlapping loci (see Supplementary Figure 1).

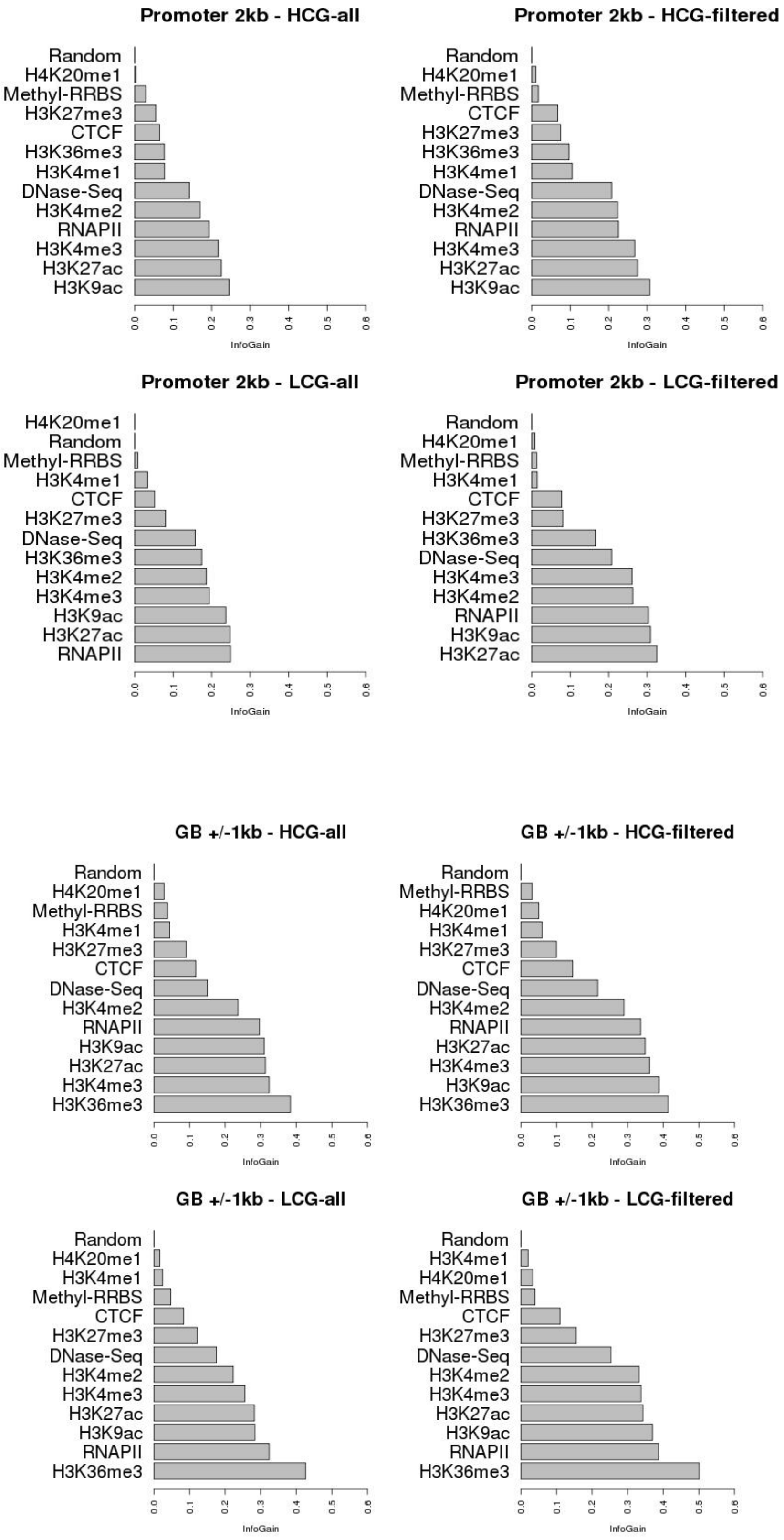

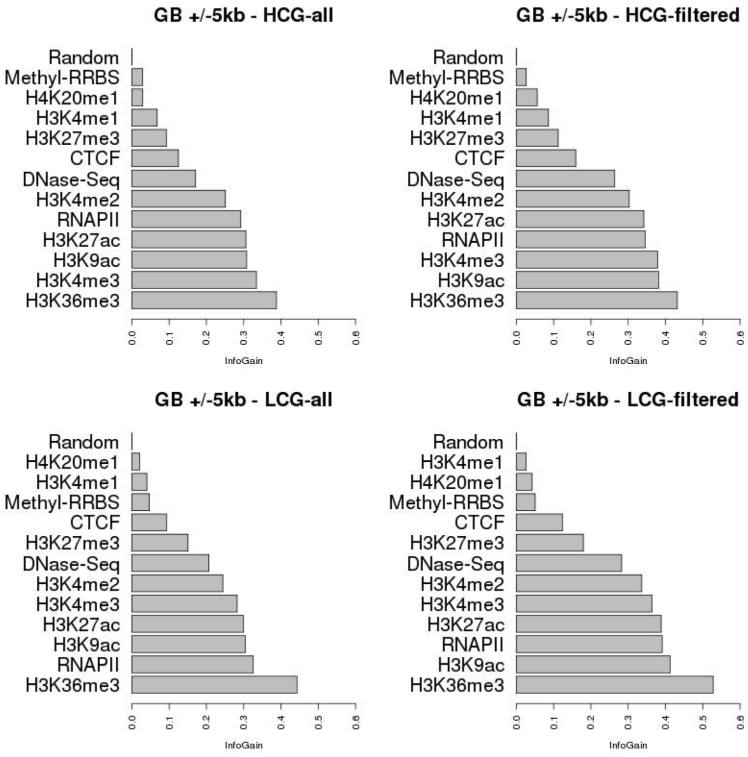
GB +/-5kb - HCG-all
Random
Methyl-RRBS
H4K20me1
H3K4me1
H3K27me3
CTCF
DNase-Seq
H3K4me2
RNAPII
H3K27ac
H3K9ac
H3K4me3
H3K36me3
0.0 0.1 0.2 0.3 0.4 0.5 0.6
InfoGain
GB +/-5kb - HCG-filtered
Random
Methyl-RRBS
H4K20me1
H3K4me1
H3K27me3
CTCF
DNase-Seq
H3K4me2
H3K27ac
RNAPII
H3K4me3
H3K9ac
H3K36me3
0.0 0.1 0.2 0.3 0.4 0.5 0.6
InfoGain
GB +/-5kb - LCG-all
Random
H4K20me1
H3K4me1
Methyl-RRBS
CTCF
H3K27me3
DNase-Seq
H3K4me2
H3K4me3
H3K27ac
H3K9ac
RNAPII
H3K36me3
0.0 0.1 0.2 0.3 0.4 0.5 0.6
InfoGain
GB +/-5kb - LCG-filtered
Random
H3K4me1
H4K20me1
Methyl-RRBS
CTCF
H3K27me3
DNase-Seq
H3K4me2
H3K4me3
H3K27ac
RNAPII
H3K9ac
H3K36me3
0.0 0.1 0.2 0.3 0.4 0.5 0.6
InfoGain

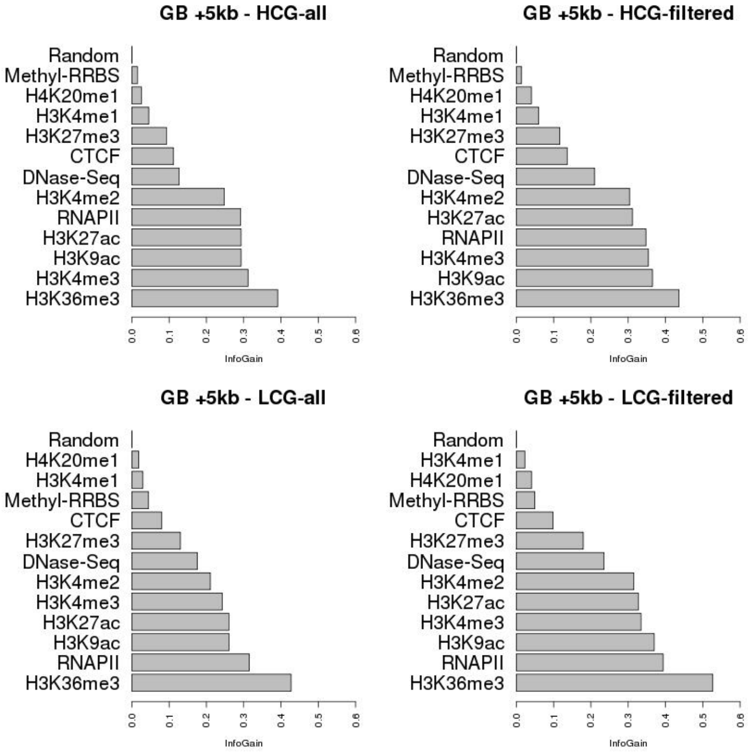
GB +5kb - HCG-all
Random
Methyl-RRBS
H4K20me1
H3K4me1
H3K27me3
CTCF
DNase-Seq
H3K4me2
RNAPII
H3K27ac
H3K9ac
H3K4me3
H3K36me3
0.0 0.1 0.2 0.3 0.4 0.5 0.6
InfoGain
GB +5kb - HCG-filtered
Random
Methyl-RRBS
H4K20me1
H3K4me1
H3K27me3
CTCF
DNase-Seq
H3K4me2
H3K27ac
RNAPII
H3K4me3
H3K9ac
H3K36me3
0.0 0.1 0.2 0.3 0.4 0.5 0.6
InfoGain
GB +5kb - LCG-all
Random
H4K20me1
H3K4me1
Methyl-RRBS
CTCF
H3K27me3
DNase-Seq
H3K4me2
H3K4me3
H3K27ac
H3K9ac
RNAPII
H3K36me3
0.0 0.1 0.2 0.3 0.4 0.5 0.6
InfoGain
GB +5kb - LCG-filtered
Random
H3K4me1
H4K20me1
Methyl-RRBS
CTCF
H3K27me3
DNase-Seq
H3K4me2
H3K27ac
H3K4me3
H3K9ac
RNAPII
H3K36me3
0.0 0.1 0.2 0.3 0.4 0.5 0.6
InfoGain

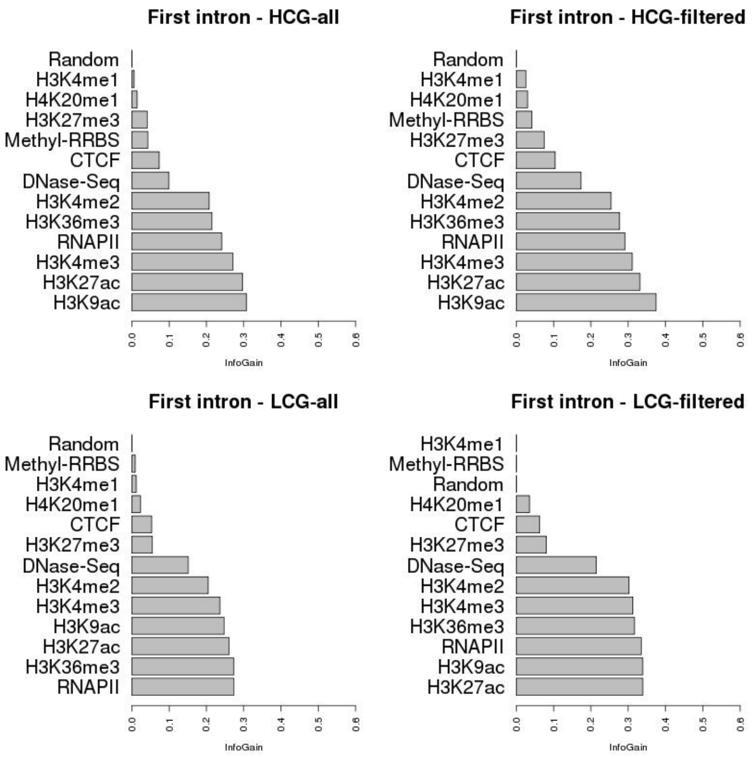
First intron - HCG-all
Random
H3K4me1
H4K20me1
H3K27me3
Methyl-RRBS
CTCF
DNase-Seq
H3K4me2
H3K36me3
RNAPII
H3K4me3
H3K27ac
H3K9ac
0.0 0.1 0.2 0.3 0.4 0.5 0.6
InfoGain
First intron - HCG-filtered
Random
H3K4me1
H4K20me1
Methyl-RRBS
H3K27me3
CTCF
DNase-Seq
H3K4me2
H3K36me3
RNAPII
H3K4me3
H3K27ac
H3K9ac
0.0 0.1 0.2 0.3 0.4 0.5 0.6
InfoGain
First intron - LCG-all
Random
Methyl-RRBS
H3K4me1
H4K20me1
CTCF
H3K27me3
DNase-Seq
H3K4me2
H3K4me3
H3K9ac
H3K27ac
H3K36me3
RNAPII
0.0 0.1 0.2 0.3 0.4 0.5 0.6
InfoGain
First intron - LCG-filtered
H3K4me1
Methyl-RRBS
Random
H4K20me1
CTCF
H3K27me3
DNase-Seq
H3K4me2
H3K4me3
H3K36me3
RNAPII
H3K9ac
H3K27ac
0.0 0.1 0.2 0.3 0.4 0.5 0.6
InfoGain

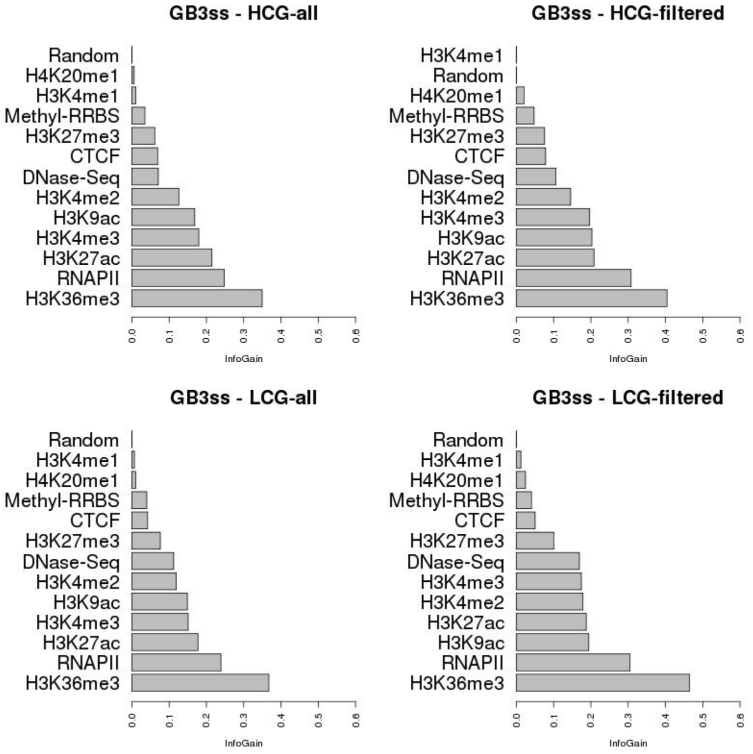
GB3ss - HCG-all
Random
H4K20me1
H3K4me1
Methyl-RRBS
H3K27me3
CTCF
DNase-Seq
H3K4me2
H3K9ac
H3K4me3
H3K27ac
RNAPII
H3K36me3
0.0 0.1 0.2 0.3 0.4 0.5 0.6
InfoGain
GB3ss - HCG-filtered
H3K4me1
Random
H4K20me1
Methyl-RRBS
H3K27me3
CTCF
DNase-Seq
H3K4me2
H3K4me3
H3K9ac
H3K27ac
RNAPII
H3K36me3
0.0 0.1 0.2 0.3 0.4 0.5 0.6
InfoGain
GB3ss - LCG-all
Random
H3K4me1
H4K20me1
Methyl-RRBS
CTCF
H3K27me3
DNase-Seq
H3K4me2
H3K9ac
H3K4me3
H3K27ac
RNAPII
H3K36me3
0.0 0.1 0.2 0.3 0.4 0.5 0.6
InfoGain
GB3ss - LCG-filtered
Random
H3K4me1
H4K20me1
Methyl-RRBS
CTCF
H3K27me3
DNase-Seq
H3K4me3
H3K4me2
H3K27ac
H3K9ac
RNAPII
H3K36me3
0.0 0.1 0.2 0.3 0.4 0.5 0.6
InfoGain

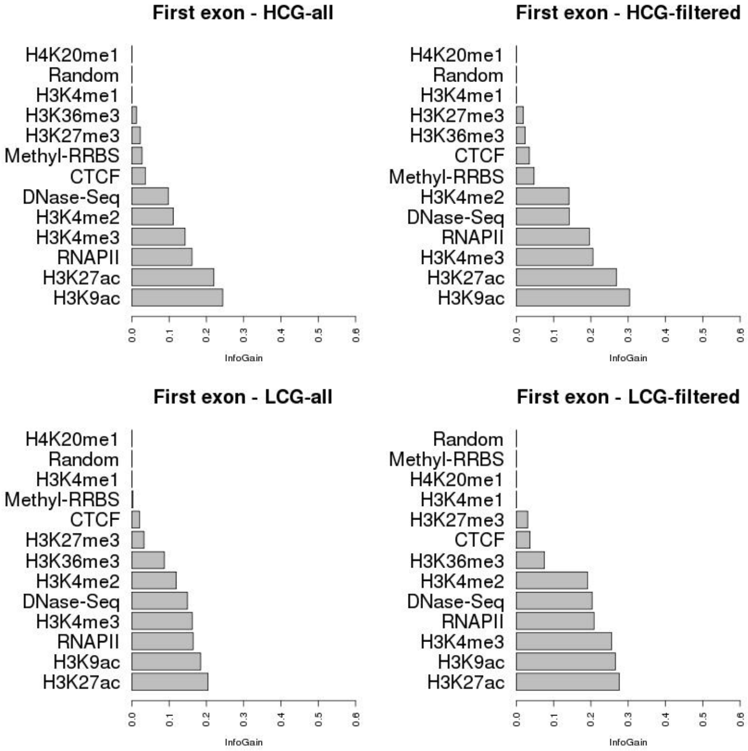
First exon - HCG-all
H4K20me1
Random
H3K4me1
H3K36me3
H3K27me3
Methyl-RRBS
CTCF
DNase-Seq
H3K4me2
H3K4me3
RNAPII
H3K27ac
H3K9ac
0.0
0.1
0.2
0.3
0.4
0.5
0.6
InfoGain
First exon - HCG-filtered
H4K20me1
Random
H3K4me1
H3K27me3
H3K36me3
CTCF
Methyl-RRBS
H3K4me2
DNase-Seq
RNAPII
H3K4me3
H3K27ac
H3K9ac
InfoGain
First exon - LCG-all
H4K20me1
Random
H3K4me1
Methyl-RRBS
CTCF
H3K27me3
H3K36me3
H3K4me2
DNase-Seq
H3K4me3
RNAPII
H3K9ac
H3K27ac
InfoGain
First exon - LCG-filtered
Random
Methyl-RRBS
H4K20me1
H3K4me1
H3K27me3
CTCF
H3K36me3
H3K4me2
DNase-Seq
RNAPII
H3K4me3
H3K9ac
H3K27ac
InfoGain

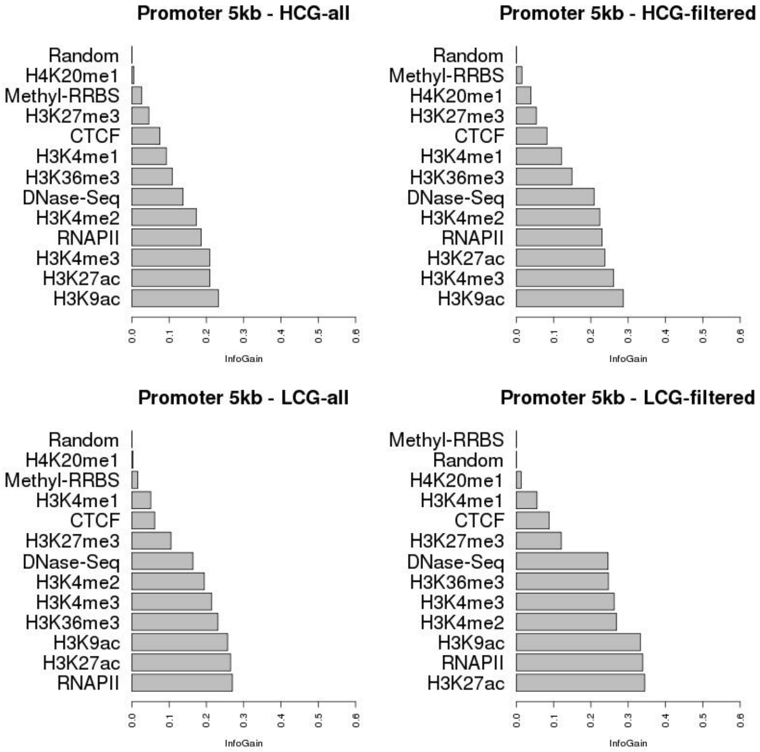
Promoter 5kb - HCG-all
Random
H4K20me1
Methyl-RRBS
H3K27me3
CTCF
H3K4me1
H3K36me3
DNase-Seq
H3K4me2
RNAPII
H3K4me3
H3K27ac
H3K9ac
0.0
0.1
0.2
0.3
0.4
0.5
0.6
InfoGain
Promoter 5kb - HCG-filtered
Random
Methyl-RRBS
H4K20me1
H3K27me3
CTCF
H3K4me1
H3K36me3
DNase-Seq
H3K4me2
RNAPII
H3K27ac
H3K4me3
H3K9ac
InfoGain
Promoter 5kb - LCG-all
Random
H4K20me1
Methyl-RRBS
H3K4me1
CTCF
H3K27me3
DNase-Seq
H3K4me2
H3K4me3
H3K36me3
H3K9ac
H3K27ac
RNAPII
InfoGain
Promoter 5kb - LCG-filtered
Methyl-RRBS
Random
H4K20me1
H3K4me1
CTCF
H3K27me3
DNase-Seq
H3K36me3
H3K4me3
H3K4me2
H3K9ac
RNAPII
H3K27ac
InfoGain

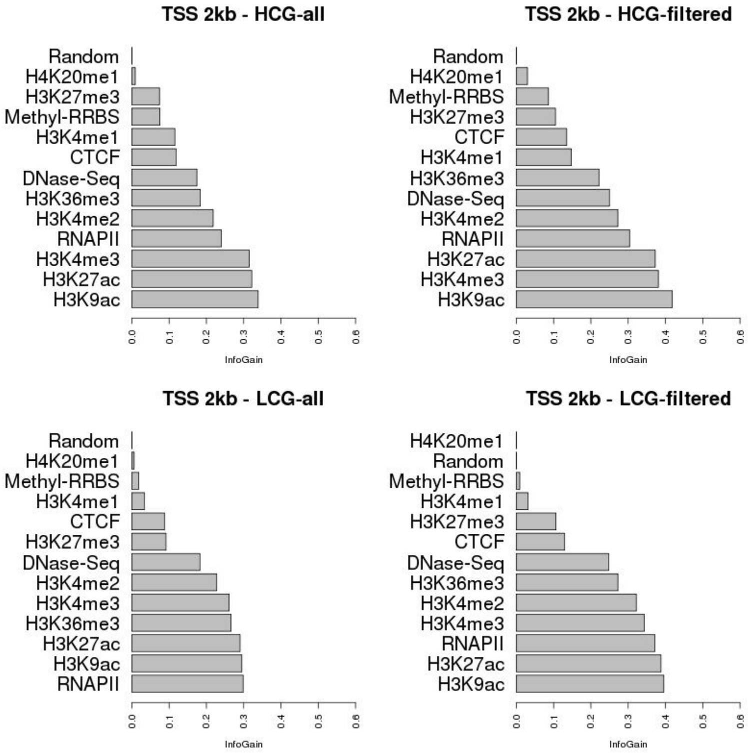
TSS 2kb - HCG-all
Random
H4K20me1
H3K27me3
Methyl-RRBS
H3K4me1
CTCF
DNase-Seq
H3K36me3
H3K4me2
RNAPII
H3K4me3
H3K27ac
H3K9ac
0.0 0.1 0.2 0.3 0.4 0.5 0.6
InfoGain
TSS 2kb - HCG-filtered
Random
H4K20me1
Methyl-RRBS
H3K27me3
CTCF
H3K4me1
H3K36me3
DNase-Seq
H3K4me2
RNAPII
H3K27ac
H3K4me3
H3K9ac
0.0 0.1 0.2 0.3 0.4 0.5 0.6
InfoGain
TSS 2kb - LCG-all
Random
H4K20me1
Methyl-RRBS
H3K4me1
CTCF
H3K27me3
DNase-Seq
H3K4me2
H3K4me3
H3K36me3
H3K27ac
H3K9ac
RNAPII
0.0 0.1 0.2 0.3 0.4 0.5 0.6
InfoGain
TSS 2kb - LCG-filtered
H4K20me1
Random
Methyl-RRBS
H3K4me1
H3K27me3
CTCF
DNase-Seq
H3K36me3
H3K4me2
H3K4me3
RNAPII
H3K27ac
H3K9ac
0.0 0.1 0.2 0.3 0.4 0.5 0.6
InfoGain

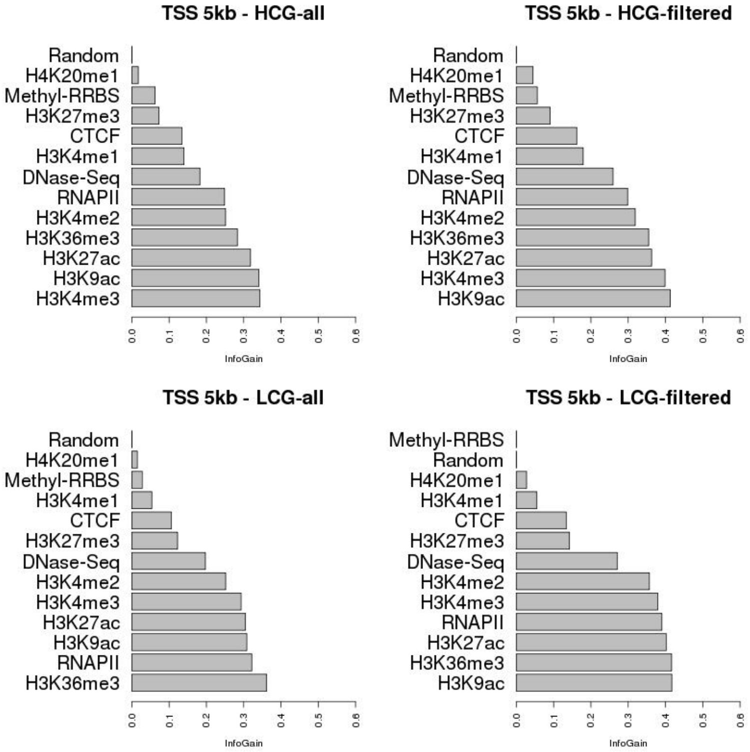
TSS 5kb - HCG-all
Random
H4K20me1
Methyl-RRBS
H3K27me3
CTCF
H3K4me1
DNase-Seq
RNAPII
H3K4me2
H3K36me3
H3K27ac
H3K9ac
H3K4me3
0.0 0.1 0.2 0.3 0.4 0.5 0.6
InfoGain
TSS 5kb - HCG-filtered
Random
H4K20me1
Methyl-RRBS
H3K27me3
CTCF
H3K4me1
DNase-Seq
RNAPII
H3K4me2
H3K36me3
H3K27ac
H3K4me3
H3K9ac
0.0 0.1 0.2 0.3 0.4 0.5 0.6
InfoGain
TSS 5kb - LCG-all
Random
H4K20me1
Methyl-RRBS
H3K4me1
CTCF
H3K27me3
DNase-Seq
H3K4me2
H3K4me3
H3K27ac
H3K9ac
RNAPII
H3K36me3
0.0 0.1 0.2 0.3 0.4 0.5 0.6
InfoGain
TSS 5kb - LCG-filtered
Methyl-RRBS
Random
H4K20me1
H3K4me1
CTCF
H3K27me3
DNase-Seq
H3K4me2
H3K4me3
RNAPII
H3K27ac
H3K36me3
H3K9ac
0.0 0.1 0.2 0.3 0.4 0.5 0.6
InfoGain

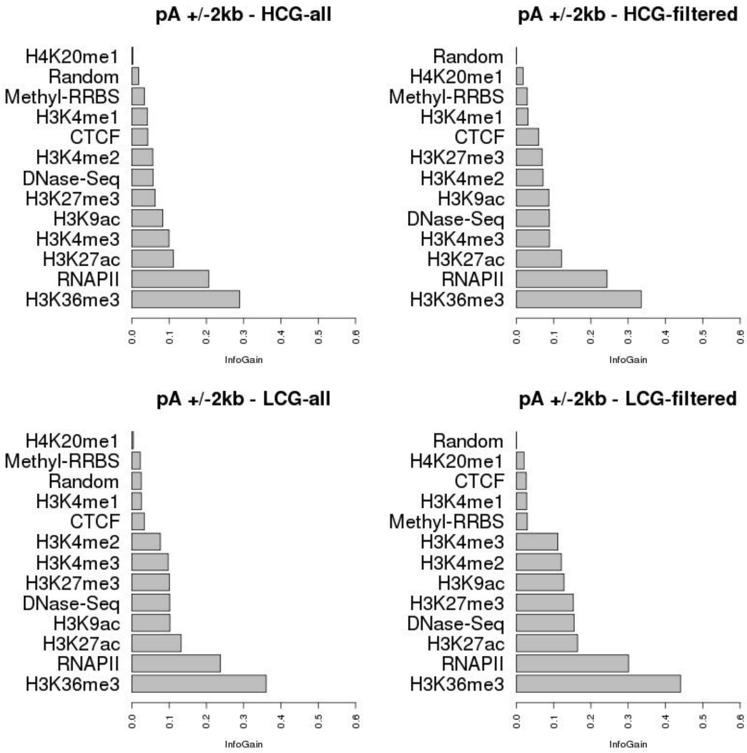
pA +/-2kb - HCG-all
H4K20me1
Random
Methyl-RRBS
H3K4me1
CTCF
H3K4me2
DNase-Seq
H3K27me3
H3K9ac
H3K4me3
H3K27ac
RNAPII
H3K36me3
0.0 0.1 0.2 0.3 0.4 0.5 0.6
InfoGain
pA +/-2kb - HCG-filtered
Random
H4K20me1
Methyl-RRBS
H3K4me1
CTCF
H3K27me3
H3K4me2
H3K9ac
DNase-Seq
H3K4me3
H3K27ac
RNAPII
H3K36me3
0.0 0.1 0.2 0.3 0.4 0.5 0.6
InfoGain
pA +/-2kb - LCG-all
H4K20me1
Methyl-RRBS
Random
H3K4me1
CTCF
H3K4me2
H3K4me3
H3K27me3
DNase-Seq
H3K9ac
H3K27ac
RNAPII
H3K36me3
0.0 0.1 0.2 0.3 0.4 0.5 0.6
InfoGain
pA +/-2kb - LCG-filtered
Random
H4K20me1
CTCF
H3K4me1
Methyl-RRBS
H3K4me3
H3K4me2
H3K9ac
H3K27me3
DNase-Seq
H3K27ac
RNAPII
H3K36me3
0.0 0.1 0.2 0.3 0.4 0.5 0.6
InfoGain

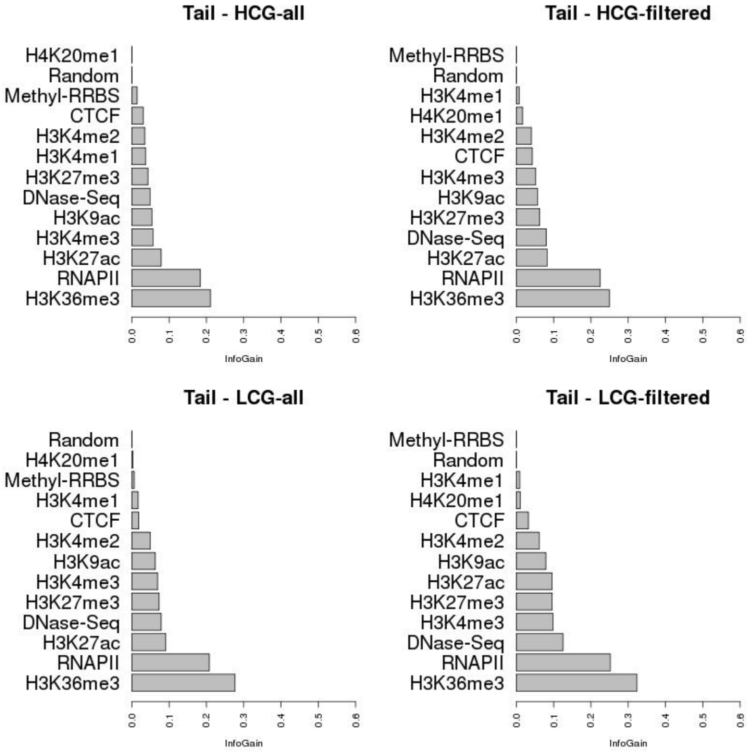
Tail - HCG-all
H4K20me1
Random
Methyl-RRBS
CTCF
H3K4me2
H3K4me1
H3K27me3
DNase-Seq
H3K9ac
H3K4me3
H3K27ac
RNAPII
H3K36me3
0.0 0.1 0.2 0.3 0.4 0.5 0.6
InfoGain
Tail - HCG-filtered
Methyl-RRBS
Random
H3K4me1
H4K20me1
H3K4me2
CTCF
H3K4me3
H3K9ac
H3K27me3
DNase-Seq
H3K27ac
RNAPII
H3K36me3
0.0 0.1 0.2 0.3 0.4 0.5 0.6
InfoGain
Tail - LCG-all
Random
H4K20me1
Methyl-RRBS
H3K4me1
CTCF
H3K4me2
H3K9ac
H3K4me3
H3K27me3
DNase-Seq
H3K27ac
RNAPII
H3K36me3
0.0 0.1 0.2 0.3 0.4 0.5 0.6
InfoGain
Tail - LCG-filtered
Methyl-RRBS
Random
H3K4me1
H4K20me1
CTCF
H3K4me2
H3K9ac
H3K27ac
H3K27me3
H3K4me3
DNase-Seq
RNAPII
H3K36me3
0.0 0.1 0.2 0.3 0.4 0.5 0.6
InfoGain

Supplementary Figure 6: DNA methylation measured in the promoter regions (2kb) of active and silent HCG transcript loci. The analysis was done in replica 1 of K562. Silent transcript loci are the 31,347 HCG transcript loci with an RPKM of 0 from RNA-Seq. Accordingly, we selected the 31,347 top scoring HCG transcript loci in terms of RPKM from RNA-Seq as active transcript loci. Silent and active loci show significantly different DNA methylation in the promoter.

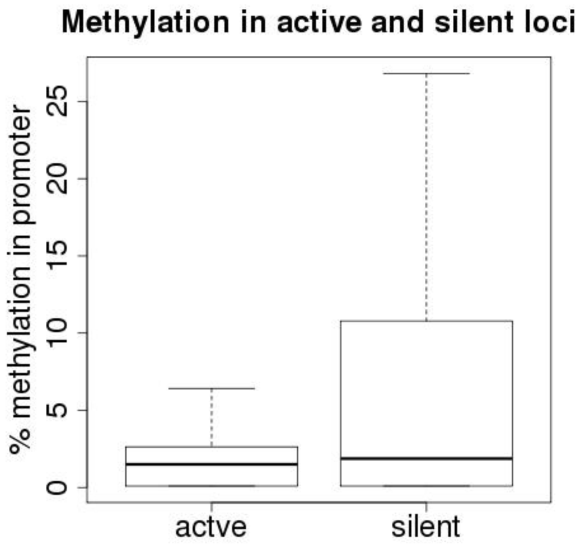

Supplementary Figure 7:

A) Distribution of z-scores for up- (Up), down- (Dw) and non- (Nr) regulated genes for the non-optimal attributes for each experiment. Optimal attributes are calculated by maximizing the Information Gain (InfoGain) (> 0.05) and minimizing the absolute value of the median for the z-score distribution of the Nr subset (< 0.1). As shown below, not all experiments have such an optimal attribute. For some experiments, the best fulfils the condition of the median for the Nr distribution, but has very low InfoGain (upper panels and H4K20me1 in the lower panel). Alternatively, some attributes have a very high InfoGain but the condition on the median is not fulfilled (RNAPII in GB3ss, lower panel). The y-axis shows the z-score corresponding to the enrichment of the attribute. These distributions correspond to the set of LCG-IC loci of Pair1.

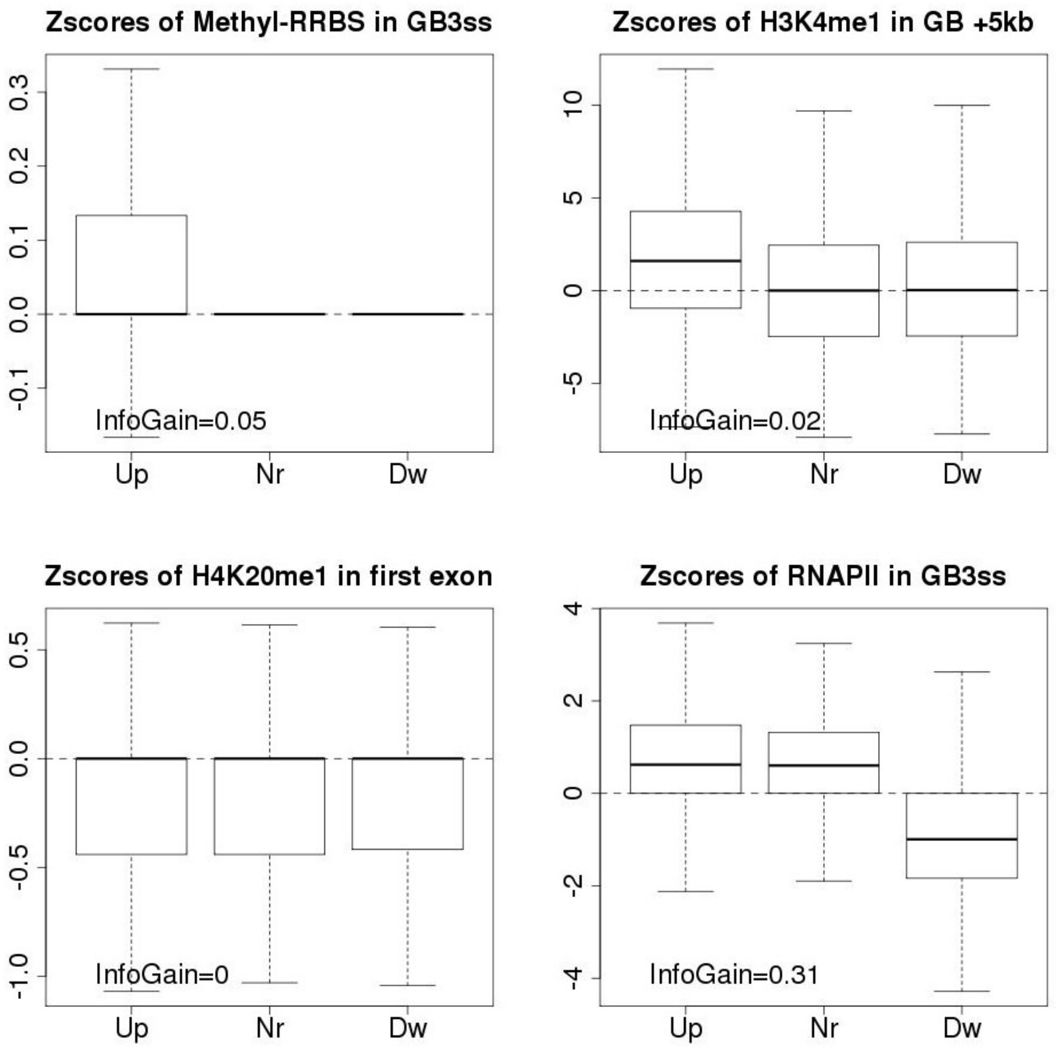

B) Overall distribution of signal from K562 and GM12878. For experiments like RNAPII, we find that the best attribute according to Information Gain (IG) has a distribution for Nr genes not centred around zero and quite similar to Up genes (See Supplementary Figure 7A). This may be due to an excess of RNAPII reads in one of the cell lines. Below we show the distribution of the signals (Percentage methylation and RPKMs) for various experiments for the same set of genes, separately for the cell lines K562 and Gm12878. The box plots show similar distributions for DNA methylation (left upper panel) and for H3K27me3 (left lower panel). However, H3K4me1 in GB +/-5kb, H4K20me1 in first exon (center and right uppper panels) and RNAPII in GB3ss (right lower panel), show an overall bias in RPKM values. This bias may be responsible for the effects observed in Supplementary Figure 7A.

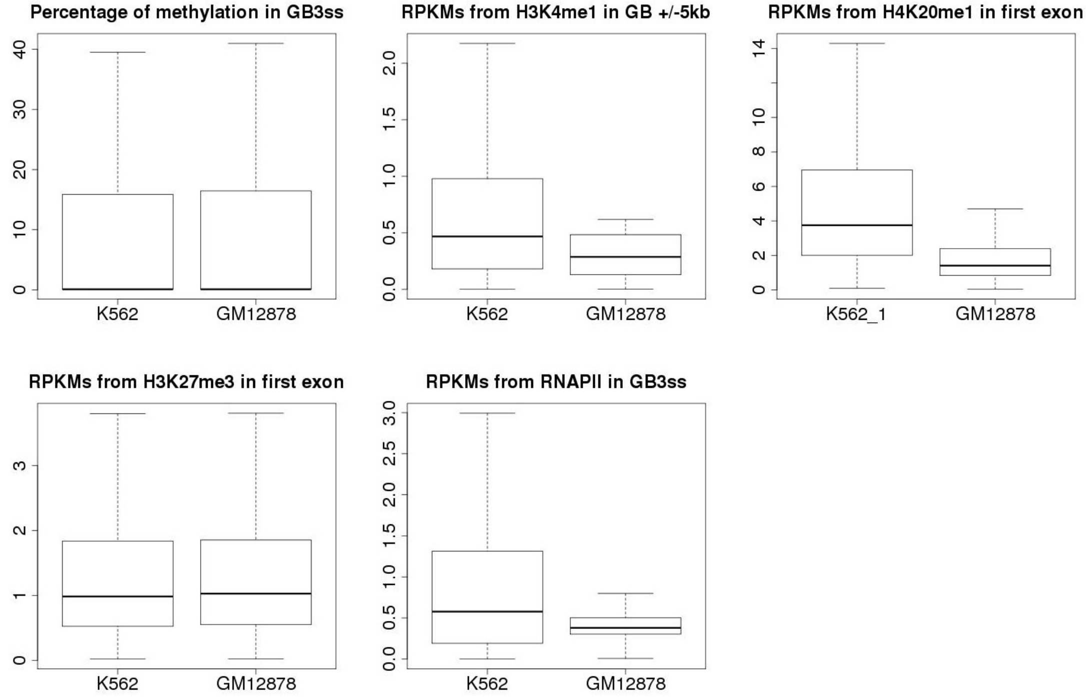

Supplementary Figure 8: Comparison of Information Gain rankings for intron-containing (IC) and intron-less (L) genes (pair 1). For this analysis we did not filtered overlapping loci, as this would lead to very small groups. Sets from Pair1 and Pair2 were selected such that they have comparable size and similar length distributions, in order to avoid unbalanced training and length biases.

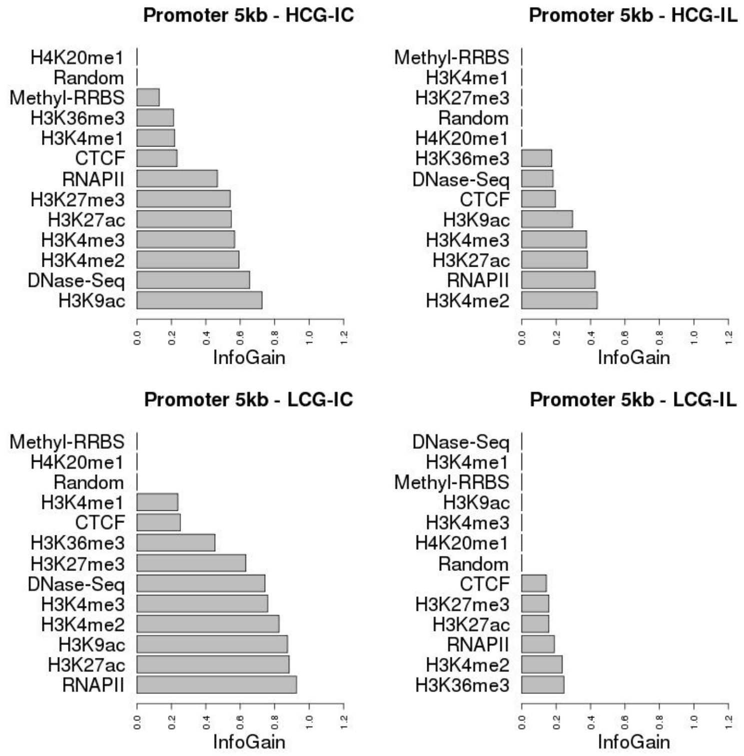

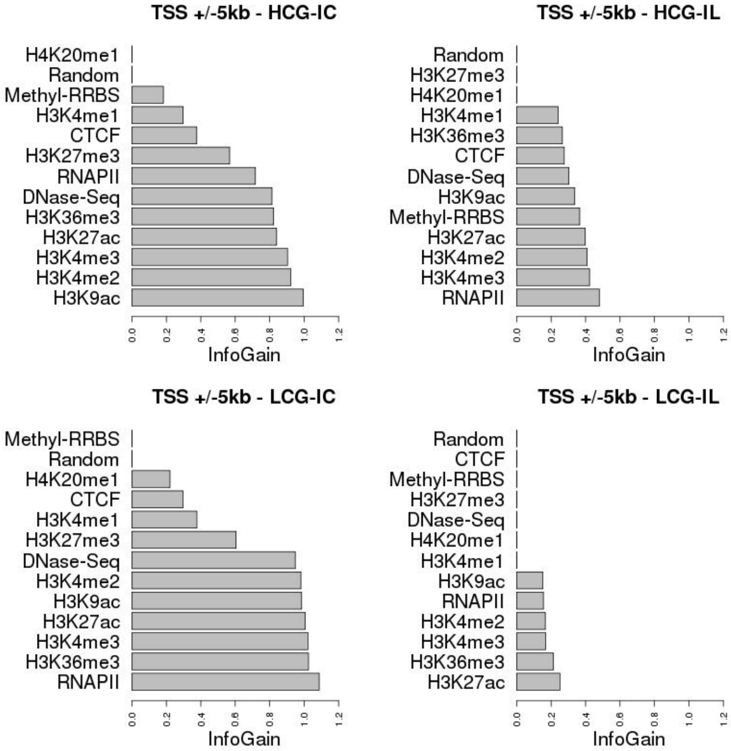
TSS +/-5kb - HCG-IC
H4K20me1
Random
Methyl-RRBS
H3K4me1
CTCF
H3K27me3
RNAPII
DNase-Seq
H3K36me3
H3K27ac
H3K4me3
H3K4me2
H3K9ac
0.0 0.2 0.4 0.6 0.8 1.0 1.2
InfoGain
TSS +/-5kb - HCG-IL
Random
H3K27me3
H4K20me1
H3K4me1
H3K36me3
CTCF
DNase-Seq
H3K9ac
Methyl-RRBS
H3K27ac
H3K4me2
H3K4me3
RNAPII
0.0 0.2 0.4 0.6 0.8 1.0 1.2
InfoGain
TSS +/-5kb - LCG-IC
Methyl-RRBS
Random
H4K20me1
CTCF
H3K4me1
H3K27me3
DNase-Seq
H3K4me2
H3K9ac
H3K27ac
H3K4me3
H3K36me3
RNAPII
0.0 0.2 0.4 0.6 0.8 1.0 1.2
InfoGain
TSS +/-5kb - LCG-IL
Random
CTCF
Methyl-RRBS
H3K27me3
DNase-Seq
H4K20me1
H3K4me1
H3K9ac
RNAPII
H3K4me2
H3K4me3
H3K36me3
H3K27ac
0.0 0.2 0.4 0.6 0.8 1.0 1.2
InfoGain

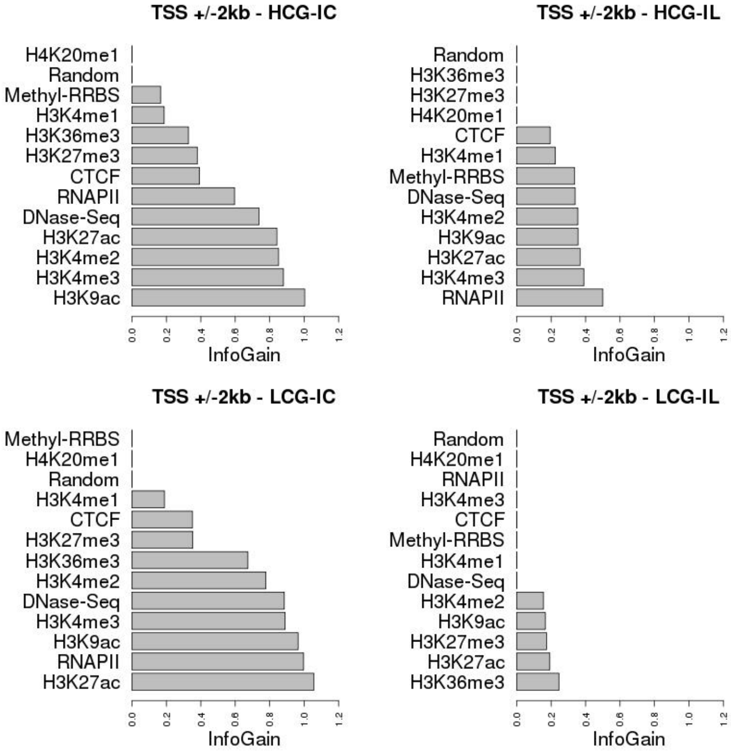
TSS +/-2kb - HCG-IC
H4K20me1
Random
Methyl-RRBS
H3K4me1
H3K36me3
H3K27me3
CTCF
RNAPII
DNase-Seq
H3K27ac
H3K4me2
H3K4me3
H3K9ac
0.0 0.2 0.4 0.6 0.8 1.0 1.2
InfoGain
TSS +/-2kb - HCG-IL
Random
H3K36me3
H3K27me3
H4K20me1
CTCF
H3K4me1
Methyl-RRBS
DNase-Seq
H3K4me2
H3K9ac
H3K27ac
H3K4me3
RNAPII
0.0 0.2 0.4 0.6 0.8 1.0 1.2
InfoGain
TSS +/-2kb - LCG-IC
Methyl-RRBS
H4K20me1
Random
H3K4me1
CTCF
H3K27me3
H3K36me3
H3K4me2
DNase-Seq
H3K4me3
H3K9ac
RNAPII
H3K27ac
0.0 0.2 0.4 0.6 0.8 1.0 1.2
InfoGain
TSS +/-2kb - LCG-IL
Random
H4K20me1
RNAPII
H3K4me3
CTCF
Methyl-RRBS
H3K4me1
DNase-Seq
H3K4me2
H3K9ac
H3K27me3
H3K27ac
H3K36me3
0.0 0.2 0.4 0.6 0.8 1.0 1.2
InfoGain

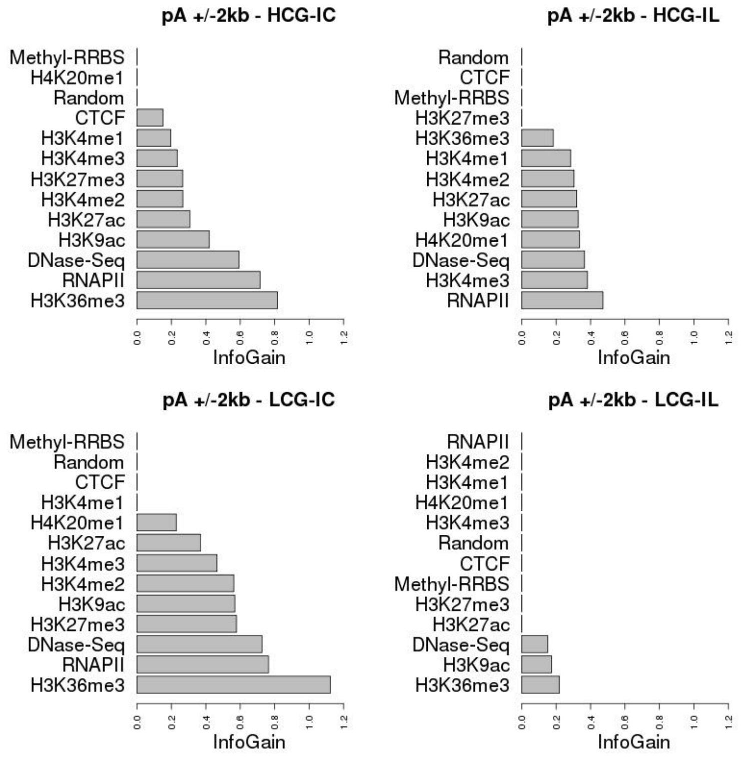
pA +/-2kb - HCG-IC
Methyl-RRBS
H4K20me1
Random
CTCF
H3K4me1
H3K4me3
H3K27me3
H3K4me2
H3K27ac
H3K9ac
DNase-Seq
RNAPII
H3K36me3
0.0
0.2
0.4
0.6
0.8
1.0
1.2
InfoGain
pA +/-2kb - HCG-IL
Random
CTCF
Methyl-RRBS
H3K27me3
H3K36me3
H3K4me1
H3K4me2
H3K27ac
H3K9ac
H4K20me1
DNase-Seq
H3K4me3
RNAPII
0.0
0.2
0.4
0.6
0.8
1.0
1.2
InfoGain
pA +/-2kb - LCG-IC
Methyl-RRBS
Random
CTCF
H3K4me1
H4K20me1
H3K27ac
H3K4me3
H3K4me2
H3K9ac
H3K27me3
DNase-Seq
RNAPII
H3K36me3
0.0
0.2
0.4
0.6
0.8
1.0
1.2
InfoGain
pA +/-2kb - LCG-IL
RNAPII
H3K4me2
H3K4me1
H4K20me1
H3K4me3
Random
CTCF
Methyl-RRBS
H3K27me3
H3K27ac
DNase-Seq
H3K9ac
H3K36me3
0.0
0.2
0.4
0.6
0.8
1.0
1.2
InfoGain

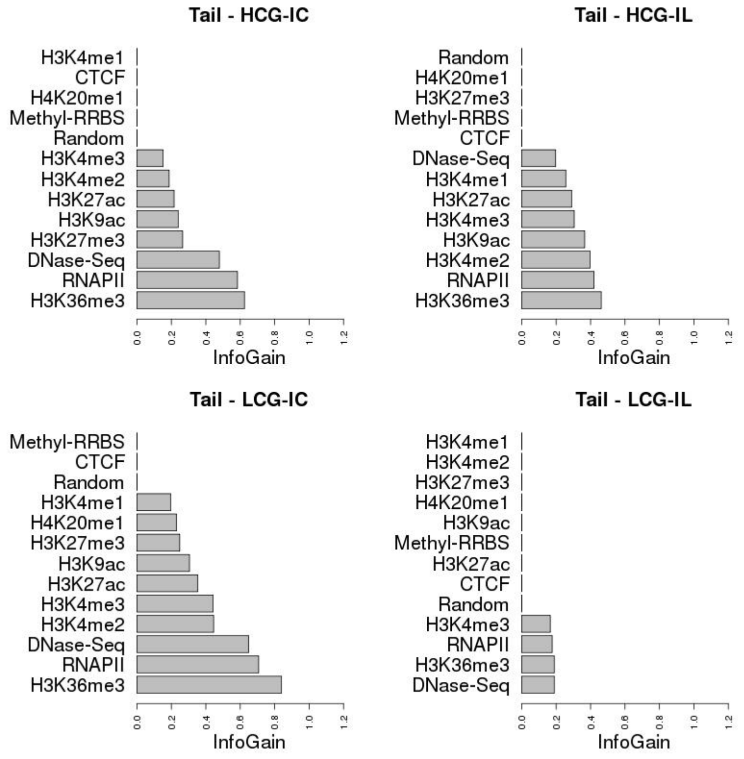
Tail - HCG-IC
H3K4me1
CTCF
H4K20me1
Methyl-RRBS
Random
H3K4me3
H3K4me2
H3K27ac
H3K9ac
H3K27me3
DNase-Seq
RNAPII
H3K36me3
0.0
0.2
0.4
0.6
0.8
1.0
1.2
InfoGain
Tail - HCG-IL
Random
H4K20me1
H3K27me3
Methyl-RRBS
CTCF
DNase-Seq
H3K4me1
H3K27ac
H3K4me3
H3K9ac
H3K4me2
RNAPII
H3K36me3
0.0
0.2
0.4
0.6
0.8
1.0
1.2
InfoGain
Tail - LCG-IC
Methyl-RRBS
CTCF
Random
H3K4me1
H4K20me1
H3K27me3
H3K9ac
H3K27ac
H3K4me3
H3K4me2
DNase-Seq
RNAPII
H3K36me3
0.0
0.2
0.4
0.6
0.8
1.0
1.2
InfoGain
Tail - LCG-IL
H3K4me1
H3K4me2
H3K27me3
H4K20me1
H3K9ac
Methyl-RRBS
H3K27ac
CTCF
Random
H3K4me3
RNAPII
H3K36me3
DNase-Seq
0.0
0.2
0.4
0.6
0.8
1.0
1.2
InfoGain

Supplementary tables:

Supplementary table 1: Accuracy in terms of the area under the ROC curve (AUC) for the 10-fold cross validation for IL gene sets under various training conditions. P1 (with RNAPII) corresponds to pair P1 with the additional RNAPII feature, i.e. the same features as P2 plus RNAPII. P1 and P2 denote the models for each cell line pairs with all the features. P1(CFS) and P2(CFS) denote the models for P1 and P2, respectively, where the features used are those that have a score of 80 or higher (maximum 100) using the CFS feature selection method independently for P1 and P2. P2 (CFS-P1) indicates that the model was trained using the data from P2 but the features selected using CFS on P1.

	HCG – IL				***LCG – IL***			
	Up	Dw	Nr	Average	Up	Dw	Nr	Average
P1 (with RNAPII)	0.88	0.91	0.87	0.88	0.72	0.7	0.71	0.71
P1	0.87	0.87	0.82	0.85	0.78	0.76	0.62	0.72
P1 (CFS)	0.87	0.91	0.84	0.87	0.81	0.7	0.65	0.72

Supplementary Table2: Features selected by Correlation Feature Selection (appearing in at least 80% of the cross validations)

A) 29 features from P1 HCG-IC before filtering ambiguous signal (bold ones also for P2)

9(90 %)	first exon Dnase
9(90 %)	first exon H3k4me2
10(100 %)	first exon H3k9ac
8(80 %)	GB3ss H3k27ac
10(100 %)	**GB3ss H3k36me3**
8(80 %)	GB +/-1kb H3k27me3
10(100 %)	GB +/-1kb H3k36me3
10(100 %)	**GB +/-5kb H3k27ac**
9(90 %)	**GB +/-5kb H3k36me3**
9(90 %)	GB +/-5kb H3k4me2
9(90 %)	**GB +5kb H3k4me3**
9(90 %)	**GB H3k27ac**
10(100 %)	**GB H3k36me3**
10(100 %)	GB H3k9ac
9(90 %)	**first intron Methyl**
9(90 %)	first intron Ctcf
8(80 %)	first intron H3k36me3
8(80 %)	**first intron H3k4me3**
8(80 %)	first intron H3k9ac
9(90 %)	Promoter 2kb H3k4me2
9(90 %)	**tail H3k36me3**
8(80 %)	TSS +/-2kb H3k4me2
9(90 %)	**TSS +/-2kb H3k9ac**
10(100 %)	**TSS +/-5kb H3k27ac**
8(80 %)	**TSS +/-5kb H3k36me3**
8(80 %)	TSS +/-5kb H3k9ac
9(90 %)	TSS +/-5kb H4K20me1
9(90 %)	**pA +/-2kb H3k27me3**
10(100 %)	**pA +/-2kb H3k36me3**

B) 33 features from P2 HCG-IC before filtering ambiguous signal (bold ones also for P1):

9(90 %)	first exon H3k4me3
9(90 %)	**GB3ss H3k36me3**
8(80 %)	GB +/-1kb H3k27ac
10(100 %)	GB +/-1kb H3k36me3
8(80 %)	GB +/-1kb H3k4me3
8(80 %)	GB +/-5kb Dnase
10(100 %)	**GB +/-5kb H3k27ac**
10(100 %)	**GB +/-5kb H3k36me3**
8(80 %)	GB +/-5kb H4K20me1
8(80 %)	GB +5kb Ctcf
8(80 %)	GB +5kb H3k36me3
9(90 %)	**GB +5kb H3k4me3**
9(90 %)	GB +5kb H4K20me1
8(80 %)	**GB H3k27ac**
10(100 %	**GB H3k36me3**
10(100 %)	GB H3k4me3
8(80 %)	**first intron Methyl**
8(80 %)	first intron H3k4me2
10(100 %)	**first intron H3k4me3**
9(90 %)	Promoter 2kb Ctcf
9(90 %)	Promoter 5kb Dnase
8(80 %)	Promoter 5kb H3k27ac
9(90 %)	**tail H3k36me3**
10(100 %)	TSS +/-2kb _H3k27ac

10(100 %)	TSS +/-2kb H3k4me3
9(90 %)	**TSS +/-2kb H3k9ac**
9(90 %)	**TSS +/-5kb H3k27ac**
10(100 %)	TSS +/-5kb H3k27me3
9(90 %)	**TSS +/-5kb H3k36me3**
10(100 %)	TSS +/-5kb H3k4me2
10(100 %)	TSS +/-5kb H3k4me3
9(90 %)	**pA +/-2kb H3k27me3**
10(100 %)	**pA +/-2kb H3k36me3**

C) 25 features from P1 LCG-IC before filtering ambiguous signal (bold ones also for P2)

9(90 %)	first exon Dnase
8(80 %)	**first exon H3k4me2**
8(80 %)	first exon H3k9ac
10(100 %)	**GB3ss H3k36me3**
8(80 %)	GB +/-1kb H3k27ac
8(80 %)	GB +/-1kb H3k27me3
10(100 %)	**GB +/-1kb H3k36me3**
10(100 %)	**GB +/-5kb H3k36me3**
8(80 %)	GB +5kb H3k9ac
10(100 %)	GB Methyl
10(100 %)	**GB H3k36me3**
8(80 %)	GB H3k9ac
8(80 %)	first intron Methyl
8(80 %)	**first intron H3k36me3**
9(90 %)	first intron H4K20me1
9(90 %)	Promoter 2kb H3k27me3
8(80 %)	Promoter 2kb H3k36me3
8(80 %)	Promoter 5kb Ctcf
9(90 %)	Promoter 5kb H3k36me3
8(80 %)	tail H3k27me3
9(90 %)	**tail H3k36me3**
8(80 %)	**TSS +/-2kb H3k36me3**
9(90 %)	TSS +/-2kb H3k9ac
10(100 %)	**TSS +/-5kb H3k36me3**
10(100 %)	**pA +/-5kb H3k36me3**

D) 20 features from P2 LCG-IC before filtering ambiguous signal (bold ones also for P1):

8(80 %)	first exon H3k36me3
8(80 %)	**first exon H3k4me2**
8(80 %)	first exon H3k4me3
10(100 %)	**GB3ss H3k36me3**
10(100 %)	**GB +/-1kb H3k36me3**
8(80 %)	**GB +/-5kb H3k36me3**
8(80 %)	GB H3k27ac
10(100 %)	**GB H3k36me3**
10(100 %)	**first intron H3k36me3**
8(80 %)	first intron H3k4me2
8(80 %)	Promoter 2kb Ctcf
8(80 %)	Promoter 5kb H3k27ac
10(100 %)	**tail H3k36me3**
8(80 %)	tail H3k9ac
10(100 %)	**TSS +/-2kb H3k36me3**
10(100 %)	TSS +/-2kb H3k4me3
9(90 %)	**TSS +/- 5kb H3k36me3**
8(80 %)	TSS +/-5kb H3k4me2
9(90 %)	pA +/-2kb H3k27me3
10(100 %)	**pA +/-2kb H3k36me3**

E) 16 features from P1 HCG-IC after filtering ambiguous signal (bold ones also appear for P2)

10(100 %)	first exon H3k4me2
8(80 %)	first exon H3k9ac
8(80 %)	**GB3ss H3k36me3**
8(80 %)	**GB +/-1kb H3k36me3**
9(90 %)	**GB +/-5kb H3k36me3**
8(80 %)	GB +/-5kb_onlyTTS H3k4me1
8(80 %)	first intron Methyl
8(80 %)	**first intron H3k27ac**
10(100 %)	Promoter 2kb H3k4me3
9(90 %)	tail H3k36me3
8(80 %)	TSS +/- 2kb Methyl
8(80 %)	TSS +/- 2kb H3k36me3
9(90 %)	**TSS +/- 5kb H3k27ac**
10(100 %)	TSS +/- 5kb H3k36me3
8(80 %)	**TSS +/- 5kb H3k4me3**
9(90 %)	pA +/- 2kb H3k36me3

F) 16 features from P2 HCG-IC after filtering ambiguous signal (bold ones also appear for P1)

8(80 %)	**GB3ss H3k36me3**
8(80 %)	GB +/-1kb H3k27a
9(90 %)	**GB +/-1kb H3k36me3**
9(90 %)	GB +/-1kb H3k4me3
8(80 %)	GB +/-5kb H3k27ac
9(90 %)	**GB +/-5kb H3k36me3**
8(80 %)	GB +/-5kb_onlyTTS H3k36me3
8(80 %)	genebody H3k36me3
9(90 %)	genebody H3k9ac
8(80 %)	**first intron H3k27ac**
10(100 %)	first intron H3k4me3
8(80 %)	first intron H3k9ac
9(90 %)	Promoter 5kb H3k27ac
10(100 %)	TSS +/- 2kb H3k4me3
8(80 %)	**TSS +/- 5kb H3k27ac**
10(100 %)	**TSS +/- 5kb H3k4me3**

G) 23 features from P1 LCG-IC after filtering ambiguous signal (bold ones also appear for P2)

10(100 %)	first exon Dnase
10(100 %)	first exon H3k4me2
10(100 %)	first exon H3k4me3
9(90 %)	first exon H3k9ac
10(100 %)	**GB3ss H3k36me3**
8(80 %)	GB3ss H3k4me3
8(80 %)	GB +/-1kb Methyl
8(80 %)	GB +/-1kb Dnase
8(80 %)	GB +/-1kb H3k27me3
9(90 %)	**GB +/-1kb H3k36me3**
8(80 %)	GB +/-1kb H3k4me2
8(80 %)	GB +/-5kb Ctcf
10(100 %)	**GB +/-5kb H3k36me3**
8(80 %)	GB +/-5kb_onlyTTS Methyl
8(80 %)	GB +/-5kb_onlyTTS Dnase
10(100 %)	**GB +/-5kb_onlyTTS H3k36me3**
9(90 %)	genebody Methyl
9(90 %)	first intron H3k27ac
8(80 %)	Promoter 2kb Ctcf
8(80 %)	Promoter 5kb H3k36me3
8(80 %)	**tail H3k36me3**
9(90 %)	**TSS +/- 5kb H3k36me3**

10(100 %) pA +/- 2kb H3k36me3

H) 13 features from P2 LCG-IC after filtering ambiguous signal (bold ones also appear for P1)

10(100 %) **GB3ss H3k36me3**
9(90 %) **GB +/-1kb H3k36me3**
9(90 %) **GB +/-5kb H3k36me3**
9(90 %) **GB +/-5kb_onlyTTS H3k36me3**
8(80 %) genebody Ctcf
9(90 %) Promoter 2kb H3k27me3
10(100 %) Promoter 2kb H3k4me3
8(80 %) **tail H3k36me3**
9(90 %) **TSS +/- 5kb H3k36me3**

Supplementary Table 3:

Accuracy on P3 in terms of the area under the ROC curve (AUC) for the 10-fold cross validation for the IL transcript sets for various training conditions**.** P3 corresponds to the fold-cross validation in pair P3. P1-on-P3 indicates that the model was trained with pair P1 and tested on pair P3. No RNAPII data was used in this analysis.

	HCG – IC				LCG – IC			
	Up	Dw	Nr	Average	Up	Dw	Nr	Average
**Before filtering**								
P3	0.74	0.76	0.66	0.72	0.79	0.82	0.66	0.76
P1-on-P3	0.68	0.73	0.59	0.67	0.74	0.77	0.58	0.7
**After filtering**								
P3	0.83	0.85	0.8	0.83	0.88	0.9	0.87	0.88
P1-on-P3	0.7	0.78	0.53	0.67	0.7	0.88	0.62	0.74

Supplementary Table 4: Feature selection per region. Considering only the attributes corresponding to a give region for prediction, we performed CFS as before. In this table we show the features that were selected with a frequency of 80% or higher from both pairs

(black), only from pair 1 (red) or only from pair 2 (blue). This analysis is done on intron containing genes for each region separately.

LCG – IC

	exon1	GB3ss	GB+/-1kb	GB+/-5kb	GB+5kb	GB	intron1	Prom 2kb	Prom 5kb	Tail	TSS+/-2kb	TSS+/-5kb	pA+/-2kb
Methyl		Methyl	Methyl		Methyl	Methyl	Methyl	Methyl					
Ctcf													CTCF
Dnase	Dnase	Dnase		Dnase	Dnase	Dnase		Dnase	Dnase	Dnase	Dnase		
H3K27ac	H3K27ac	H3K27ac	H3K27ac	H3K27ac	H3K27ac	H3K27ac	H3K27ac	H3K27ac	H3K27ac	H3K27ac	H3K27ac	H3K27ac	H3K27ac
H3K27me3	H3K27me3	H3K27me3	H3K27me3	H3K27me3	H3K27me3	H3K27me3	H3K27me3	H3K27me3	H3K27me3	H3K27me3	H3K27me3	H3K27me3	H3K27me3
H3K36me3	H3K36me3	H3K36me3	H3K36me3	H3K36me3	H3K36me3	H3K36me3	H3K36me3	H3K36me3	H3K36me3	H3K36me3	H3K36me3	H3K36me3	H3K36me3
H3K4me1								H3K4me1	H3K4me1	H3K4me1			
H3K4me2	H3K4me2		H3K4me2			H3K4me2	H3K4me2	H3K4me2	H3K4me2		H3K4me2	H3K4me2	
H3K4me3	H3K4me3	H3K4me3	H3K4me3	H3K4me3	H3K4me3		H3K4me3	H3K4me3	H3K4me3		H3K4me3	H3K4me3	
H3K9ac	H3K9ac	H3K9ac	H3K9ac	H3K9ac	H3K9ac	H3K9ac	H3K9ac	H3K9ac	H3K9ac		H3K9ac	H3K9ac	
H4K20me1													

HCG - IC

Methyl	exon1	GB3ss	GB+/-1kb	GB+/-5kb	GB+5kb	GB	intron1	Prom 2kb	Prom 5kb	Tail	TSS+/-2kb	TSS+/-5kb	pA+/-2kb
Ctcf													
Dnase	Dnase		Dnase	Dnase		Dnase	Dnase	Dnase	Dnase	Dnase	Dnase	Dnase	
H3K27ac	H3K27ac	H3K27ac	H3K27ac	H3K27ac	H3K27ac	H3K27ac	H3K27ac	H3K27ac	H3K27ac	H3K27ac	H3K27ac	H3K27ac	
H3K27me3		H3K27me3		H3K27me3	H3K27me3			H3K27me3	H3K27me3	H3K27me3	H3K27me3	H3K27me3	
H3K36me3		H3K36me3	H3K36me3	H3K36me3	H3K36me3	H3K36me3	H3K36me3		H3K36me3	H3K36me3	H3K36me3	H3K36me3	H3K36me3
H3K4me1	H3K4me1							H3K4me1	H3K4me1			H3K4me1	
H3K4me2	H3K4me2		H3K4me2	H3K4me2	H3K4me2	H3K4me2	H3K4me2	H3K4me2	H3K4me2		H3K4me2	H3K4me2	
H3K4me3	H3K4me3	H3K4me3	H3K4me3	H3K4me3	H3K4me3	H3K4me3	H3K4me3	H3K4me3	H3K4me3		H3K4me3	H3K4me3	
H3K9ac	H3K9ac	H3K9ac	H3K9ac	H3K9ac	H3K9ac	H3K9ac	H3K9ac	H3K9ac	H3K9ac		H3K9ac	H3K9ac	
H4K20me1													

Supplementary Table 5: Accuracy of predictions using only features that are selected from both pair 1 and pair 2. The accuracy is given in terms of the area under the ROC curve (AUC). Predictions are done on pair 2 in intron containing genes for each region separately. The attributes used are those from Supplementary Table 4 that are selected from both pairs after CFS, with frequency >=80%.

LCG-IC -using only intersecting attributes (black)

	exon1	GB3ss	GB+/-1kb	GB+/-5kb	GB+5kb	GB	intron1	Prom 2kb	Prom 5kb	Tail	TSS+/-2kb	TSS+/-5kb	pA+/-2kb	all
UP	0.84	0.88	0.92	0.92	0.91	0.91	0.89	0.88	0.91	0.89	0.93	0.92	0.85	0.86
DW	0.82	0.79	0.91	0.91	0.88	0.88	0.86	0.86	0.89	0.82	0.92	0.89	0.78	0.89
nonReg	0.73	0.73	0.86	0.86	0.84	0.82	0.81	0.76	0.81	0.74	0.83	0.84	0.69	0.76
average	0.8	0.8	0.9	0.9	0.88	0.87	0.85	0.83	0.87	0.82	0.89	0.88	0.78	0.84

HCG-IC -using only intersecting attributes (black)

	exon1	GB3ss	GB+/-1kb	GB+/-5kb	GB+5kb	GB	intron1	Prom 2kb	Prom 5kb	Tail	TSS+/-2kb	TSS+/-5kb	pA+/-2kb	all
UP	0.79	0.82	0.87	0.88	0.88	0.86	0.85	0.77	0.78	0.76	0.86	0.88	0.81	0.79
DW	0.79	0.76	0.85	0.86	0.86	0.86	0.82	0.77	0.77	0.75	0.86	0.88	0.72	0.82
nonReg	0.76	0.7	0.81	0.8	0.82	0.81	0.79	0.67	0.7	0.66	0.82	0.83	0.69	0.75
average	0.78	0.76	0.84	0.85	0.85	0.84	0.82	0.73	0.75	0.72	0.85	0.86	0.74	0.79

Supplementary methods:

Command lines for Pyicos (version 1.0.3) enrichment analysis:

For ChIP-Seq and Dnase-Seq:

```
pyicos enrichment wgEncodeBroadHistoneK562H3k9acStdAlnRep1.filtered.sam
wgEncodeBroadHistoneGm12878H3k9acStdAlnRep1.filtered.sam RESULT_pA +/-
2kb__k562_Gm12878__H3k9ac.enrichment -o -f sam --replica-a
wgEncodeBroadHistoneK562H3k9acStdAlnRep2.filtered.sam -o --region pA +/- 2kb.bed
--region-format bed --n-norm --len-norm --binstep 1000 --pseudocount
```

For DNA methylation data (mean methylation with 0.1 as pseudocount):

```
pyicos enrichcount K562_Gm_meth_genebody.mean.pc RESULT_K562_Gm_meth_genebody.mean.enr
--total-reads-a 10000000 --total-reads-b 10000000
```

total reads do not matter in this case as we do not normalize

For RNA-Seq (RPKMs from ENCODE with 3.5e-5 as pseudocount (half of minimum in K562_1)):

```
pyicos enrichcount RPKM.K562_Gm12878 RESULT_RPKM_K562_Gm12878.enr --total-reads-a
10000000 --total-reads-b 10000000
```

total reads do not matter in this case as we do not normalize

Biomart-powered database

We used Biomart [1] as the platform for deploying the enrichment data set between different cell lines used in our analysis. Each database includes a number of datasets, one per each pair of cell lines compared in terms of enrichment. We populated the Biomart database with enrichment z-scores for the datasets from Table 2. These are stored as a set of feature tables, where each feature is a pair (signal,region), .e.g. RNAPII-genebody or H3K27me3-TSS +/- 2kb, built from the datasets from Table 2 and the regions described in Table 3 of the manuscript. Together with this Biomart database, we installed a local mirror of Ensembl Biomart (Release 62) [2] in order to make crossed queries possible between our set of databases and the Ensembl Release 62 Mart Database. Additionally, we modified Biomart so that data sets can be exported as ARFF (attribute-relation file format), which can be uploaded directly into the WEKA system [3], providing flexibility for training models for the study of mechanisms of gene regulation.

Our databases are accessible at http://regulatorygenomics.upf.edu/group/pages/software .

Bibliography

[1] A. Kasprzyk, "BioMart: driving a paradigm change in biological data management," *Database: The Journal of Biological Databases and Curation*, vol. 2011, p. bar049, 2011.

[2] P. Flicek et al., "Ensembl 2012," *Nucleic Acids Research*, vol. 40, no. Database issue, pp. D84-90, Jan. 2012.

[3] Frank E, Hall M, Trigg L, Holmes G, Witten IH. Data mining in bioinformatics using Weka. Bioinformatics. 2004 Oct 12;20(15):2479–81.
